# Effects of Curcumin Supplementation on Production Performance, Intestinal Health and Cecal Microbiota in Late-Phase Jingfen No. 1 Laying Hens

**DOI:** 10.3390/ani16213415

**Published:** 2026-10-29

**Authors:** Haoliang Chai, Dexin Zhao, Taibiao Wang, Guanyu Hou, Liguang Shi, Maoyuan Wang, Euphrème Ipemba, Lessebe Gambou Dieu Leveut, Hanfeng Li, Hongzhi Wu, Ting Cao

**Affiliations:** 1 Tropical Crops Genetic Resources Institute, Chinese Academy of Tropical Agricultural Sciences, Haikou 571101, China; 2 College of Animal Science and Technology, Northeast Agricultural University, Harbin 150000, China; 3 College of Animal Science and Technology, Henan University of Science and Technology, Luoyang 471023, China; 4 National Centre for Crop Disease Control, Ministry of Agriculture, Animal Husbandry and Fisheries, Brazzaville 999059, Congo; 5 China-Aid-Congo (B) Agricultural Technology Demonstration Center, Brazzaville 999059, Congo

**Keywords:** curcumin, late-phase laying hens, cecal microbiota, oxidative stress, intestinal barrier

## Abstract

This study investigated the effects of graded dietary curcumin supplementation on production performance, antioxidant capacity, immune status, intestinal barrier function, and cecal microbiota in late-laying hens. A total of 480 61-week-old Jingfen No. 1 laying hens were randomly allocated to four groups with six replicates of 20 birds each: a basal diet control (CG) and diets supplemented with 100, 200, or 400 mg/kg curcumin (CN1, CN2 and CN3) for 35 days. Curcumin supplementation significantly increased average egg weight (*p* < 0.05). Serum catalase concentration and immunoglobulin levels (IgM, IgA, and IgY) were elevated, whereas malondialdehyde, interleukin-6, interleukin-10, and tumor necrosis factor-α levels were reduced in a dose-dependent manner (*p* < 0.05). Similar improvements were observed in the jejunum, including increased glutathione peroxidase and catalase concentrations and reduced inflammatory cytokines. Birds in the CN3 group exhibited greater villus height and villus-to-crypt ratio, lower crypt depth, and upregulated mRNA expression of tight junction proteins (*ZO-1*, *Occludin*, and *Claudin-1*) (*p* < 0.05). Curcumin also modulated the cecal microbiota by enriching beneficial butyrate-producing bacteria (*Lactobacillales*, *Faecalibacterium*, *Odoribacter*, *Roseburia*, and *Phascolarctobacterium*) and reducing pathogenic *Staphylococcus* and *Bilophila*. Overall, dietary curcumin improved egg quality, antioxidant and immune functions, intestinal health, and microbial composition in late-laying hens.

## Simple Summary

As laying hens grow older and enter the late laying period, their physical condition and egg production gradually decline. They are prone to oxidative stress, inflammation, and impaired intestinal health, which adversely affect their overall condition and egg quality. Since antibiotics are no longer ideal choices for feed use, researchers are looking for safe natural additives for poultry farming instead. This study evaluated the effects of different dietary levels of curcumin, a natural plant extract, in older laying hens. The results showed that dietary curcumin reduced oxidative damage and inflammation and enhanced immune responses in the hens. It also improved intestinal structure and strengthened the intestinal barrier. Curcumin also altered the cecal bacterial community, increasing beneficial bacteria and reducing harmful bacteria. Among all tested doses, 400 mg per kilogram of feed produced the most consistent overall responses. These findings suggest that curcumin may be a promising natural feed additive. Curcumin may improve the health of late-phase laying hens and help maintain production performance, although further studies are needed to confirm its value under commercial farming conditions.

## 1. Introduction

Laying hens undergo concurrent deterioration in productive performance and physiological health during the late laying stage [1]. After peak lay, laying rate and egg quality decline, and these changes are closely associated with impaired intestinal function [2]. The intestinal microbiota changes throughout the laying cycle, with distinct community compositions during the early, peak, and late laying phases. The intestine serves as a vital organ responsible for nutrient digestion, absorption and immune defense in laying hens [3]. Hens aging into the late laying stage commonly exhibit compromised intestinal barrier integrity. Structural damage, deterioration of intestinal morphology, and impaired barrier function are major intestinal health problems in aged laying hens [4].

Late-phase laying hens also exhibit increased oxidative stress and systemic inflammation. Oxidative damage accumulates progressively after the peak laying period, accompanied by increased expression of pro-inflammatory cytokines such as IL-6 and reduced antioxidant enzyme activity. These physiological disturbances further impair intestinal barrier structure and function [5]. Antibiotic administration during the laying period may raise concerns regarding egg safety and public health. Growing consumer demand for green, antibiotic-free and low-residue livestock products has driven extensive research into alternative feed additives. Plant-derived additives have emerged as promising alternatives to antibiotics and synthetic additives because of their natural origin, diverse biological activities, environmental benefits, and low residue levels. Several natural bioactive substances, including puerarin [6], quercetin [7] and silymarin [8], have been reported to effectively improve the productive performance and health status of late-phase laying hens.

Curcumin is a natural polyphenolic compound derived from the rhizome of Curcuma longa with potent antioxidant, anti-inflammatory, antibacterial, and immunomodulatory properties [9,10]. It has attracted increasing research interest as a sustainable feed additive in poultry nutrition. Numerous studies have demonstrated that dietary curcumin supplementation effectively optimizes intestinal morphology and immune function in laying hens. Supplemental curcumin at doses of 100 to 400 mg/kg improves intestinal villus height and upregulates the gene expression of digestive enzymes in hens during the early laying stage [11]. Such dietary intervention also increases antioxidant enzyme concentrations and immunoglobulin synthesis. The upregulated expression of intestinal tight junction proteins further confirms the positive regulatory role of curcumin in maintaining intestinal barrier integrity. In studies focusing on peak-phase breeding hens, the same dosage range of curcumin significantly reduces the abundance of the detrimental genus Barnesiella and enriches beneficial Lactobacillus populations [12]. Overall, curcumin improves intestinal health in laying hens by improving intestinal morphology, reinforcing barrier integrity, and reshaping intestinal microbial communities, making it a promising alternative to antibiotic feed additives in commercial poultry production.

Jingfen No. 1 is a pink-shell laying hen strain independently bred by Beijing Huadu Yukou Poultry Industry Co., Ltd., characterized by strong adaptability, high egg yield and superior egg quality [13]. Despite the well-documented beneficial effects of curcumin on laying hen physiology, existing studies have focused largely on the early and peak laying stages. Limited information is available regarding the efficacy of curcumin supplementation for improving the health and performance of hens in the late laying phase. Accordingly, the present study used 61-week-old Jingfen No. 1 hens at the late laying stage as experimental animals. Graded concentrations of curcumin were added to the basal diet to evaluate changes in cecal microbial diversity and community composition, serum and intestinal markers of oxidative stress and inflammation, intestinal tight junction gene expression, and intestinal morphology. This study aimed to provide empirical evidence to support the use of curcumin as a functional feed additive for late-laying hens and a theoretical basis for extending the productive lifespan of commercial laying hens.

## 2. Materials and Methods

### 2.1. Animal Ethics Statement

All animal husbandry and experimental procedures involved in this study were approved by the Institutional Animal Care and Use Committee of the Chinese Academy of Tropical Agricultural Sciences (approval number: CATAS20260301-3). The entire experimental workflow was conducted in strict compliance with the relevant laws and regulations of the local jurisdiction, as well as the institutional requirements.

### 2.2. Experimental Materials

Curcumin used in this study was obtained from Shaanxi Baichuan Biotechnology Co., Ltd. (Xi’an, China). Total curcuminoids accounted for 95% of the commercial product by weight. Within the total curcuminoid fraction, the relative proportions of the individual curcuminoids were 77% curcumin, 15% demethoxycurcumin, and 6% bisdemethoxycurcumin (summing to 98% of the total curcuminoid fraction, with the remaining 2% consisting of other minor curcuminoids). Curcumin and demethoxycurcumin serve as the major active ingredients. Qualitative and quantitative analysis of these bioactive components was conducted at the Chinese Academy of Tropical Agricultural Sciences (Haikou, China) via high-performance liquid chromatography coupled with mass spectrometry.

Jingfen No. 1 laying hens were used as experimental animals in the current trial. All birds were hatched and reared under standard laboratory management conditions. The formal animal trial commenced when the hens reached 61 weeks of age, corresponding to the late laying stage.

### 2.3. Experimental Design and Management

This feeding trial lasted 35 days. A total of 480 Jingfen No. 1 laying hens were stratified by pre-experimental body weight, laying rate and average egg weight at 60 weeks of age, and then randomly allocated to four dietary treatments, with six replicates per treatment and 20 hens per replicate. Baseline production performance was determined before curcumin supplementation to verify the uniformity of hens across groups (Table S1). All replicates were housed in individual cages measuring 1000 mm × 1000 mm × 500 mm. The control group (CG) received a basal diet, whereas the three treatment groups (CN1, CN2, and CN3) were fed basal diets supplemented with 100, 200 and 400 mg/kg curcumin, respectively. The dietary curcumin levels were selected based on previously published findings by Xu et al. [11] and Nawab et al. [14] and preliminary data from our research team. The replicate (cage) was considered the experimental unit for treatment effects, and individual hens were used as sampling units. For production performance, all hens in each replicate were used, and data were averaged per replicate before analysis (*n* = 6 replicates per treatment). For serum antioxidant and inflammatory parameters, one hen per replicate was randomly selected for blood collection (n = 6 replicates per treatment). For cecal microbiota analysis, one hen per replicate was randomly selected and euthanized for cecal content collection (n = 6 replicates per treatment). Technical replicates were averaged before statistical analysis and were not treated as independent biological replicates.

The basal diet was formulated to meet the nutritional requirements of laying hens, in accordance with the 10th revised edition of Nutrient Requirements of Poultry issued by the National Academies of Sciences, Engineering, and Medicine (NASEM, 2025). The detailed dietary composition and nutrient levels are presented in Table 1. Curcumin powder was uniformly incorporated into the experimental diets during feed preparation. Briefly, all conventional feed ingredients were first ground using a 9FC21 bench-top pulverizer. The pulverized raw materials were subsequently transferred into a YGH 100 rotary mixer (Shanghai Nie’er Machinery Manufacturing Co., Ltd., Shanghai, China), where precisely quantified curcumin powder was added. The mixture was blended continuously at a rotation speed of 18 rpm for 45 min to achieve homogeneous mixing. The blended feed was then processed into pellets using an RT 2S pellet mill (Henan Changxing Machinery Equipment Co., Ltd., Zhengzhou, China) [15].

Throughout the experimental period, indoor temperature was maintained at 18–24 °C and relative humidity at 50–70%. The trial was carried out in a naturally ventilated poultry house fitted with bilateral ventilation windows, with routine ventilation conducted at 08:00 every morning. A standard photoperiod of 17 h of light and 7 h of darkness was provided via 5000 K cool-white LED lamps with a light intensity of 15 lux. All experimental hens were allowed ad libitum access to pelleted feed and clean drinking water. Feed was offered in V-shaped troughs partitioned with foam boards to isolate independent replicates, which prevented hens from accessing feed intended for other replicates and allowed accurate daily measurement of residual feed. Drinking water was supplied through nipple drinkers, and regular waterline disinfection was performed once a week. Hens were fed at 07:00 and 18:00 daily to maintain consistent feeding management throughout the trial.

### 2.4. Production Data Collection

The amounts of feed offered and remaining, the number of eggs produced daily, and individual egg weights were recorded for each replicate throughout the trial. Average daily feed intake per hen was calculated based on total feed consumption of each replicate throughout the experimental period. The total feed consumption was determined as the difference between total feed offered and residual feed, with residual feed collected and weighed before each feeding. Feed consumption was then divided by the number of hens and the trial duration. Daily egg production was calculated using the total number of qualified eggs collected per replicate, which was further standardized to the number of experimental hens and trial days. The laying rate was calculated as the number of qualified eggs divided by the total number of hen-days, multiplied by 100. All calculations were performed on a hen-day basis given that no bird mortality occurred during the entire feeding period. Average egg weight was determined by dividing the total egg weight recorded per replicate by the corresponding total egg number. The feed-to-egg ratio was calculated as total feed intake divided by total egg weight for each replicate. All productive parameters were initially calculated at the replicate level before subsequent statistical analysis across experimental groups.

### 2.5. Sample Collection

All experimental hens were fasted for 12 h before sample collection, with free access to drinking water. At the end of the experiment, one hen per replicate (six hens per treatment) was randomly selected for sample collection. Birds were euthanized via jugular vein exsanguination. During slaughter, approximately 10 mL of blood was harvested from the jugular vein using anticoagulant-free vacuum tubes. Blood samples were kept at room temperature for 30 min to allow natural coagulation, followed by centrifugation at 3000 r/min for 15 min at 4 °C. The separated serum supernatant was aliquoted into cryogenic vials and stored at −80 °C for subsequent biochemical analysis.

Immediately after euthanasia, the abdominal cavity was opened to collect the duodenum, jejunum and ileum according to standard anatomical characteristics. For intestinal morphological analysis, 1–2 cm tissue segments were collected from the middle portion of the duodenum, jejunum and ileum. The collected intestinal segments were gently rinsed with pre-cooled sterile phosphate-buffered saline to clear luminal contents. Cleaned tissue samples were fixed in 4% paraformaldehyde solution at 4 °C for 48 h and then transferred to 70% ethanol for long-term preservation at 4 °C. For the quantification of intestinal tight junction protein gene expression, 3–5 cm tissue segments were harvested from the middle section of each intestinal segment. The intestinal tissues were opened longitudinally and rinsed with pre-cooled sterile phosphate-buffered saline. Intestinal mucosa was carefully scraped using the blunt edge of a sterile glass slide. The collected jejunal mucosa was mixed with pre-chilled normal saline at a mass-to-volume ratio of 1:9 and fully homogenized in an ice bath. The homogenate was centrifuged at 3000 r/min for 10 min at 4 °C, and the resulting supernatant was stored at −20 °C for subsequent determination of jejunal biochemical indices. The remaining mucosal tissues were placed in cryovials, snap-frozen in liquid nitrogen and maintained at −80 °C for further molecular analysis. For 16S rRNA gene sequencing, approximately 2–4 g of fresh cecal contents was collected into sterile centrifuge tubes. All biological samples were obtained from the same selected hen within each replicate. The cecal content samples were immediately snap-frozen in liquid nitrogen and stored at −80 °C until subsequent microbial community analysis.

### 2.6. Biochemical Indicators

Frozen serum samples and jejunal mucosal supernatants were thawed gently prior to biochemical analysis. ELISA kits from Nanjing Jiancheng Bioengineering Institute (Nanjing, China) were used to measure oxidative stress indices and immune-related indicators. The oxidative stress parameters included superoxide dismutase (SOD), glutathione peroxidase (GSH-Px), catalase (CAT), and malondialdehyde (MDA). For SOD, GSH-Px, and CAT, the ELISA results represented protein concentrations rather than enzymatic catalytic activities. The immune and inflammatory biomarkers included immunoglobulin A (IgA), immunoglobulin Y (IgY), immunoglobulin M (IgM), interleukin-6 (IL-6), interleukin-10 (IL-10), and tumor necrosis factor-alpha (TNF-α). All indicators were quantified by ELISA using a Varioskan LUX microplate reader (Thermo Fisher Scientific, Shanghai, China).

The total protein concentration of each jejunal mucosal supernatant was determined using the BCA Protein Assay Kit (Nanjing Jiancheng Bioengineering Institute, Nanjing, China; Cat. No. A045-4) according to the manufacturer’s instructions. Analyte concentrations in the jejunal mucosal supernatants were normalized to the total protein concentration of the corresponding supernatant.

For serum samples, GSH-Px was expressed as pmol/mL; SOD and MDA as pg/mL; CAT as ng/L; IgM and IgA as ng/mL; IgY as μg/mL; and IL-10, IL-6, and TNF-α as ng/L. For jejunal mucosal samples, GSH-Px was expressed as pmol/mg protein; SOD and MDA as pg/mg protein; CAT as ng/mg protein; IgM and IgA as ng/mg protein; IgY as μg/mg protein; and IL-10, IL-6, and TNF-α as ng/mg protein.

All ELISA assays were performed in strict accordance with the manufacturer’s standard protocols. Microplates were incubated at 37 °C for 60 min and rinsed three times with PBS-T buffer to eliminate residual unbound substances. Subsequent incubation with substrate solution was conducted at room temperature for 15 min. The enzymatic reaction was terminated by the addition of stop solution, and the absorbance of each well was immediately measured at 450 nm.

### 2.7. Intestinal Histological Morphology

Following tissue fixation, duodenal, jejunal, ileal samples were processed using standard histological techniques. All tissue specimens were dehydrated through a graded ethanol series, cleared with xylene and embedded in paraffin wax. Serial tissue sections 4–5 μm thick were cut using a Leica RM2255 microtome (Leica Microsystems, Wetzlar, Germany) and mounted on clean glass slides. Hematoxylin and eosin staining was conducted in line with conventional histological protocols. Briefly, paraffin sections were dewaxed in xylene and rehydrated using a descending ethanol concentration gradient. Nuclei were stained with hematoxylin, and cytoplasmic structures were counterstained with eosin. After staining, sections were dehydrated, cleared and sealed with coverslips using neutral mounting medium. Histological images were acquired using an EVOS FL Auto biological microscope (Thermo Fisher Scientific, Waltham, MA, USA). The full experimental procedures were implemented in accordance with the methods previously reported by Yu et al. [16]. For intestinal morphometric analysis, three non-consecutive sections per hen were selected for each segment, and five well-oriented villi and corresponding crypts were measured per section, giving 15 villus–crypt units per segment per hen. Only intact, vertically oriented villi with an intact lamina propria and clearly identifiable crypt openings were measured. Villus height and crypt depth were measured using ImageJ v1.53 at 100× magnification. Measurements were performed with the observer blinded to treatment allocation, and the mean of the 15 villus–crypt units per segment per hen was used for statistical analysis.

### 2.8. Cecal Content 16S rRNA Gene Sequencing

Cecal content samples preserved at −80 °C were thawed slowly in a 4 °C refrigerator. All sample tubes were maintained in an upright position throughout the process to avoid contamination of the tube caps by intestinal contents. The V4 hypervariable region of the bacterial 16S rRNA gene was amplified using the universal primers 515F (5′-GTGCCAGCMGCCGCGGTAA-3′) and 806R (5′-GGACTACHVGGGTWTCTAAT-3′). Amplified PCR products were processed through end repair, addition of a single adenine nucleotide and adapter ligation to construct sequencing libraries, which were subsequently subjected to paired-end sequencing on the Illumina NovaSeq platform. For cecal microbiota analysis, six biological samples per treatment group (*n* = 6) were analyzed. Raw sequencing reads were quality filtered using fastp (version 1.0.1) software with the following parameters: reads containing more than 5 N bases, quality score < Q19, or >15% low-quality bases were removed, and chimeric sequences were eliminated via the vsearch algorithm to obtain valid clean sequences. After quality filtering and chimera removal, no additional abundance-based taxon filtering was applied. Amplicon sequence variants were generated using the DADA2 algorithm embedded in QIIME2 (v202504), and taxonomy was assigned using the Silva 138.2 reference database. To eliminate sequencing depth bias across samples, all datasets were normalized to the minimum sequencing depth of 69,613 valid reads prior to downstream bioinformatic analysis. Alpha diversity indices, including Chao1, Shannon, Simpson, and dominance, were calculated to evaluate bacterial richness, diversity, and community dominance. Overall differences in each alpha diversity index among the four dietary groups were assessed using the Kruskal–Wallis H test. Rank-based pairwise comparisons were subsequently performed using the nonparametric multiple-comparison procedure implemented in the kruskal function of the R package agricolae. For each index, the six pairwise *p* values were adjusted using the Benjamini–Hochberg procedure, and adjusted *p* < 0.05 was considered statistically significant. Beta diversity was assessed based on weighted and unweighted UniFrac distances to characterize microbial structural variations among groups, with principal coordinates analysis (PCoA), non-metric multidimensional scaling (NMDS; stress < 0.2), and unweighted pair-group method with arithmetic mean (UPGMA) clustering. PERMANOVA (ADONIS) was performed based on Bray–Curtis distance with 999 permutations to test the significance of community compositional differences among treatments. Analysis of similarities (ANOSIM) with 999 permutations based on Bray–Curtis distance was also performed to further confirm group separation. Microbial taxa that differed among treatments were further identified using the Wilcoxon rank-sum test, the Kruskal–Wallis H test, linear discriminant analysis effect size (LEfSe), and MetagenomeSeq, along with similarity percentage (SIMPER) analysis based on Bray–Curtis dissimilarity. LEfSe was performed with an LDA score threshold of 4.0 and a significance level of *p* < 0.05. For differential taxon analyses, where applicable, *p* values were adjusted for multiple testing using the Benjamini–Hochberg false-discovery-rate (FDR) procedure, and an adjusted *q* value < 0.05 was considered statistically significant. All 16S rRNA gene sequencing and bioinformatic analyses were performed using the NovoMagic Cloud platform (https://magic.novogene.com/, accessed on 31 May 2026). Raw sequencing data generated in this study have been deposited in the National Center for Biotechnology Information Sequence Read Archive under the BioProject accession number PRJNA1468589.

### 2.9. Quantitative Real-Time PCR (qPCR)

Total RNA was isolated from approximately 100 mg of mucosal tissues sampled from the duodenum, jejunum, and ileum using the RNeasy Mini Kit (QIAGEN, Hilden, Germany). RNA integrity was assessed by 2% agarose gel electrophoresis, and RNA concentration and purity were measured using a NanoPhotometer^®^ (Implen, Munich, Germany). Only RNA samples with an A_260_/A_280_ ratio between 1.8 and 2.1 were used for subsequent experiments. Genomic DNA contamination was removed from 1 μg of total RNA that met the quality criteria using a gDNA Eraser (TaKaRa, Dalian, China). Reverse transcription was carried out using the PrimeScript^®^ RT Reagent Kit (TaKaRa) in accordance with the manufacturer’s protocols. The synthesized cDNA was stored at −20 °C for subsequent quantitative real-time polymerase chain reaction (qPCR) analysis. qPCR amplification was performed on an Applied Biosystems PRISM 7500 Fast Real-Time PCR System (Applied Biosystems, Foster City, CA, USA) with SYBR^®^ Premix Ex Taq II (TaKaRa). The 20 μL reaction mixture contained SYBR Premix, 0.4 μM of each primer, 10 ng of cDNA template, and RNase-free water. The thermal cycling protocol consisted of an initial pre-denaturation at 95 °C for 30 s, followed by 40 cycles of 95 °C for 5 s and 60 °C for 34 s. Melting curve analysis was performed by gradually increasing the temperature from 60 °C to 95 °C at an increment of 0.3 °C every 15 s to verify specific amplification.

Two stable housekeeping genes, *Act-β* (*Actin-β*) and *GAPDH* (*Glyceraldehyde-3-phosphate Dehydrogenase*), served as internal references. The expression stability of *Act-β* and *GAPDH* across all experimental samples was evaluated using geNorm, NormFinder, and BestKeeper algorithms. Both genes exhibited stable expression under the present experimental conditions (geNorm *M* < 0.5; NormFinder stability value < 0.15; BestKeeper SD < 0.5). Their geometric mean was used to normalize the expression of target genes. The target genes examined in this study included *TJP1* (*tight junction protein 1*, also known as *ZO-1*), *OCLN* (*occludin*), and *CLDN1* (*claudin 1*).

Primer amplification efficiencies were determined by generating standard curves using 10-fold serial dilutions of pooled cDNA template. The amplification efficiencies for *TJP1*, *OCLN*, *CLDN1*, *Act-β*, and *GAPDH* were 97.5%, 96.8%, 98.2%, 99.1%, and 98.6%, respectively, with *R*^2^ > 0.99 for all primer pairs. All values fell within the acceptable range of 90–110%, satisfying the assumptions of the 2^−ΔΔCt^ method. The control group (CG, 0 mg/kg curcumin) was used as the calibrator for relative expression calculation.

The relative mRNA expression levels were calculated using the 2^−ΔΔCt^ method. All primer sequences for the detected genes are listed in Table 2.

Each qPCR reaction was performed in technical triplicate to ensure the accuracy and reproducibility of the experimental data. The Ct values of technical triplicates for each biological sample were averaged prior to normalization and statistical analysis. Technical replicates were not treated as independent biological observations; only the averaged Ct value from each biological replicate was used for subsequent statistical analysis.

### 2.10. Statistical Analysis

Production performance and physiological data were analyzed using IBM SPSS 26.0 software (Armonk, NY, USA). Alpha diversity data were analyzed using the Kruskal–Wallis H test and rank-based pairwise comparisons implemented in the R package agricolae, with Benjamini–Hochberg adjustment for multiple comparisons. One-way ANOVA was performed to evaluate differences in measured parameters among dietary treatment groups. Where significant overall differences were identified, Duncan’s multiple range test was adopted for pairwise post hoc comparisons. All experimental results are presented as means with the standard error of the mean, and differences were considered statistically significant at *p* < 0.05. Orthogonal polynomial contrast analysis was further conducted to determine the linear and quadratic responses of laying hen physiological indices to graded curcumin supplementation levels. Because the curcumin doses (0, 100, 200, and 400 mg/kg) were unequally spaced, the linear and quadratic contrast coefficients were generated using the ORTHO polynomial procedure in SPSS with the actual dose values specified as level values, rather than using default equally spaced coefficients. The coefficients used were: linear = −7, −3, 1, 9; quadratic = 7, −4, −8, 5.

The experimental unit was the replicate (cage). For production performance, all hens in each replicate were used, and data were averaged per replicate before analysis (*n* = 6 replicates per treatment). For serum and jejunal mucosal antioxidant and immune/inflammatory parameters, intestinal morphology, qPCR, and cecal microbiota analysis, one hen per replicate was randomly selected for sampling, and the value obtained from this hen was treated as the value of that replicate. The replicate was used as the experimental unit for statistical analysis (*n* = 6 replicates per treatment). Individual hens originating from the same replicate were not treated as independent observations. The statistical model used in the present study was defined as Yij = μ + Ti + eij. In this formula, Yij indicates the observed value of the j-th replicate in the i-th treatment group, μ represents the overall population mean, Ti stands for the fixed treatment effect, and eij refers to the random experimental error. All experimental figures were generated using FigDraw (Home for Researchers, Hangzhou, China; www.figdraw.com, accessed on 31 May 2026).

## 3. Results

### 3.1. Production Performance

Curcumin supplementation significantly affected average egg weight (AEW; *p* = 0.031). The AEW was higher in CN3 (58.35 g) than in CG (52.17 g; *p* < 0.05), whereas no significant differences were observed in the other pairwise comparisons. AEW increased linearly with increasing dietary curcumin level (*p* = 0.008), with no significant quadratic effect (*p* = 0.364). Furthermore, the weekly production data (Table S2) revealed a time-dependent effect: AEW did not differ among groups in Week 1, but the curcumin-supplemented groups showed progressively higher AEW compared to the CG, with the differences becoming significant from Week 3 to Week 5 (*p* < 0.01). No significant effects were observed on ADFI, EPR, or FER (*p* > 0.05; Table 3).

### 3.2. Serum Antioxidant Indices

Serum MDA decreased linearly (*p* < 0.001) and CAT increased linearly (*p* < 0.001) with curcumin addition. CN3 had lower MDA (242.32 vs. 362.53 pg/mL) and higher CAT (78.64 vs. 41.79 ng/L) than CG (*p* < 0.05). GSH-Px and SOD were unaffected (*p* > 0.05; Table 4).

### 3.3. Serum Inflammatory and Immune Indices

Dietary curcumin supplementation significantly affected serum immunoglobulin and inflammatory cytokine concentrations (*p* < 0.05). Serum IgM, IgA, and IgY concentrations increased linearly with increasing curcumin level (*p* = 0.007, *p* < 0.0001, and *p* = 0.004, respectively). The IgM concentration was higher in CN3 (6004 ng/mL) than in CG (4696 ng/mL; *p* < 0.05). Serum IgA peaked in CN2 (8667 ng/mL) and was greater than in CG (6034 ng/mL; *p* < 0.05). Serum IgY was higher in CN3 (84.53 μg/mL) than in CG (62.36 μg/mL; *p* < 0.05). The concentrations of IL-10, IL-6, and TNF-α decreased linearly with increasing curcumin supplementation (*p* = 0.004, *p* = 0.012, and *p* = 0.003, respectively). The CN3 group exhibited the lowest levels of IL-10 (84.65 ng/L), IL-6 (19.97 ng/L), and TNF-α (103.41 ng/L), all lower than those in CG (*p* < 0.05). Serum IgA showed a significant quadratic response to curcumin supplementation (*p* < 0.0001), while no quadratic effects were detected for other serum immune and inflammatory indices (*p* > 0.05; Table 5).

### 3.4. Jejunal Antioxidant Indices

Jejunal GSH-Px, MDA, and CAT concentrations were significantly influenced by curcumin supplementation (*p* < 0.0001). Jejunal GSH-Px and CAT concentrations increased linearly with increasing curcumin level (*p* < 0.0001). The GSH-Px concentration was highest in CN3 (23.39 pmol/mg protein) and was greater than that in CG (16.09 pmol/mg protein; *p* < 0.05). The jejunal CAT concentration was also highest in CN3 (90.20 ng/mg protein) and was greater than that in CG (67.05 ng/mg protein; *p* < 0.05). The jejunal MDA concentration decreased linearly with curcumin supplementation (*p* < 0.0001), being lowest in CN3 (398.98 pg/mg protein) and lower than that in CG (658.18 pg/mg protein; *p* < 0.05). No significant differences were observed in jejunal SOD concentration among treatments (*p* > 0.05), and no quadratic effects were detected for jejunal antioxidant indices (*p* > 0.05; Table 6).

### 3.5. Jejunal Inflammatory and Immune Indices

Curcumin supplementation significantly affected all measured jejunal immune and inflammatory parameters except IL-6 (*p* < 0.05). Jejunal IgM, IgA, and IgY concentrations increased linearly with increasing curcumin level (*p* < 0.0001, *p* = 0.005, and *p* = 0.002, respectively). The CN3 group had the highest jejunal IgM (4172 ng/mg protein) and IgA (5013 ng/mg protein) concentrations, both of which were greater than those in CG (3109 and 4017 ng/mg protein, respectively; *p* < 0.05). Jejunal IgY concentrations were higher in all curcumin-supplemented groups than in CG (90.05 μg/mg protein; *p* < 0.05). Jejunal IL-10 and TNF-α concentrations decreased linearly with curcumin supplementation (*p* < 0.0001 and *p* = 0.007, respectively). The CN3 group had the lowest jejunal IL-10 (201.56 ng/mg protein) and TNF-α (236.58 ng/mg protein) concentrations, both of which were lower than those in CG (319.22 and 404.72 ng/mg protein, respectively; *p* < 0.05). No significant differences were observed in jejunal IL-6 concentration among treatments (*p* > 0.05). Jejunal IgY exhibited a quadratic trend that did not reach statistical significance (*p* = 0.069), while no quadratic effects were detected for the other jejunal immune and inflammatory indices (*p* > 0.05; Table 7).

### 3.6. Intestinal Morphology

Dietary curcumin supplementation significantly improved morphological parameters of the duodenum, jejunum, and ileum (*p* < 0.05). In the duodenum, villus height and villus height-to-crypt depth ratio (V/C) increased linearly with curcumin level (*p* < 0.0001), while crypt depth decreased linearly (*p* = 0.011). The CN3 group had the greatest duodenal villus height (817.68 μm) and V/C ratio (7.91), as well as the lowest crypt depth (106.80 μm); all three values differed significantly from those in the CG (*p* < 0.05). In the jejunum, villus height increased linearly (*p* = 0.007), while crypt depth and V/C ratio exhibited both linear and quadratic responses (*p* < 0.05). The CN3 group had the greatest jejunal villus height (766.11 μm; *p* < 0.05). In the ileum, villus height and V/C ratio increased linearly (*p* = 0.002 and *p* < 0.0001, respectively), while crypt depth decreased linearly (*p* < 0.0001). The CN2 and CN3 groups had greater ileal villus height and V/C ratio, as well as lower crypt depth, compared with the CG (*p* < 0.05; Table 8).

### 3.7. Intestinal Tight Junction Protein mRNA Expression

The mRNA expression levels of genes encoding intestinal tight junction proteins (*ZO-1*, *occludin*, and *claudin-1*) were significantly upregulated by curcumin supplementation (*p* < 0.05). In the duodenum, the mRNA expression of *ZO-1* and *Occludin* increased linearly (*p* = 0.002 and *p* < 0.0001, respectively), while *Claudin-1* exhibited both linear and quadratic responses (*p* = 0.016 and *p* = 0.006). The CN2 and CN3 groups had greater duodenal tight junction protein mRNA expression than CG (*p* < 0.05). In the jejunum, the mRNA expression of *ZO-1*, *Occludin*, and *Claudin-1* showed both linear and quadratic responses (*p* < 0.05), with the highest mRNA expression observed in CN2 or CN3 (*p* < 0.05). In the ileum, the mRNA expression of *ZO-1* and *Occludin* increased linearly and quadratically (*p* < 0.0001), while *Claudin-1* mRNA increased linearly (*p* < 0.0001). The CN3 group had the highest ileal tight junction protein mRNA expression, which was greater than that in the CG (*p* < 0.05; Table 9).

### 3.8. Cecal Microbial Diversity and Dominance

**Figure 1:**
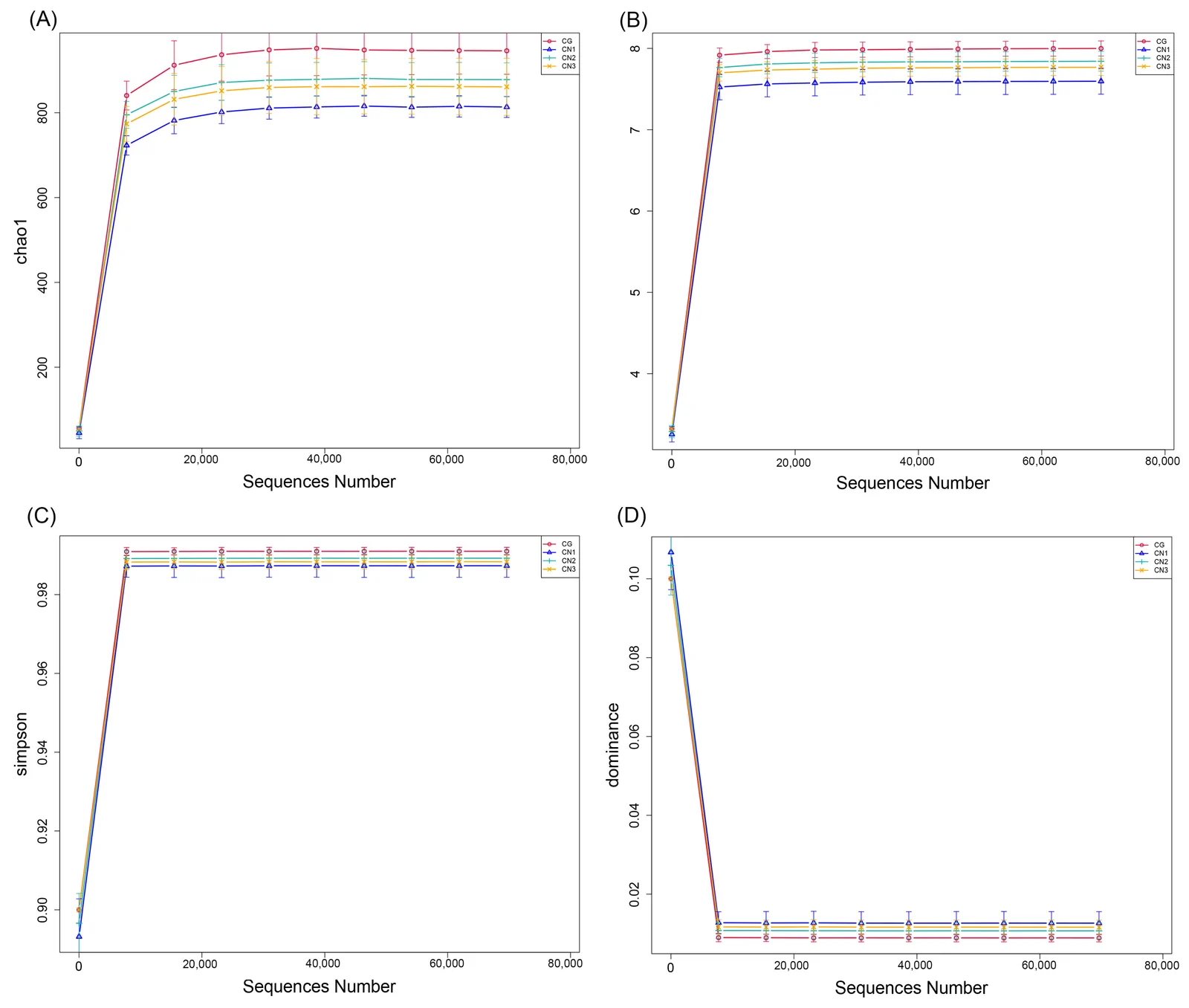
Species diversity curve. (**A**) Chao index rarefaction curve. (**B**) Shannon index rarefaction curve. (**C**) Simpson index rarefaction curve. (**D**) Dominance index rarefaction curve. CG represented the control group, whereas CN1, CN2, and CN3 were experimental groups with dietary curcumin supplementation at 100, 200, and 400 mg/kg, respectively. Six samples were analyzed per group.

**Figure 2:**
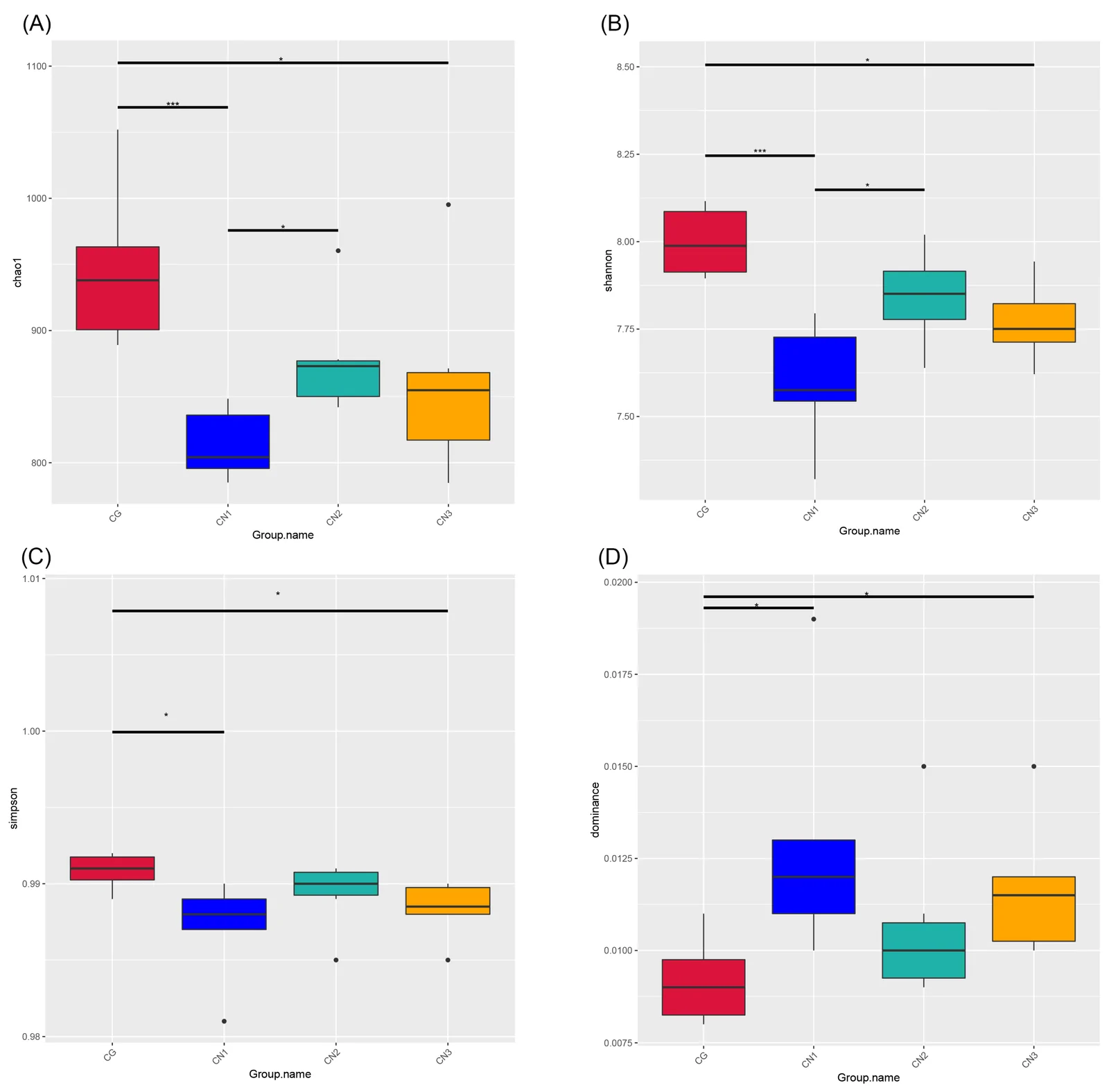
Comparison of alpha diversity indices of cecal microbiota among treatment groups. (**A**) Chao1 index. (**B**) Shannon index. (**C**) Simpson index. (**D**) Dominance index. Overall differences among groups were assessed using the Kruskal–Wallis H test, followed by rank-based pairwise comparisons with Benjamini–Hochberg adjustment within each index. Asterisks indicate significant pairwise differences based on adjusted *p* values: * adjusted *p* < 0.05, *p* < 0.01, and *** adjusted *p* < 0.001. CG represented the control group, whereas CN1, CN2, and CN3 were experimental groups with dietary curcumin supplementation at 100, 200, and 400 mg/kg, respectively. Six samples were analyzed per group.

Rarefaction curves for all alpha diversity metrics reached stable plateaus, confirming adequate sequencing depth and comprehensive coverage of the cecal microbial communities across all samples (Figure 1A–D). In this study, the Chao1 index was used to estimate bacterial community richness, whereas the Shannon and Simpson indices were adopted to evaluate community diversity. The alpha diversity of the cecal microbiota differed among treatment groups. Following rank-based pairwise comparisons with Benjamini–Hochberg adjustment, the Chao1 index was significantly higher in the CG than in the CN1 (adjusted *p* < 0.001) and CN3 groups (adjusted *p* = 0.015), whereas the difference between the CG and CN2 groups was not statistically significant (adjusted *p* = 0.056). In addition, the CN2 group showed a higher Chao1 index than the CN1 group (adjusted *p* = 0.028) (Figure 2A). The Shannon index exhibited a similar variation pattern, with higher values in the CG than in the CN1 (adjusted *p* < 0.001) and CN3 groups (adjusted *p* = 0.012), but no significant difference between the CG and CN2 groups (adjusted *p* = 0.061). The Shannon index was also higher in the CN2 group than in the CN1 group (adjusted *p* = 0.014). For the Simpson index, the CG showed significantly higher values than the CN1 (adjusted *p* = 0.016) and CN3 groups (adjusted *p* = 0.028) (Figure 2B,C). Conversely, community dominance was significantly lower in the CG than in the CN1 (adjusted *p* = 0.016) and CN3 groups (adjusted *p* = 0.028) (Figure 2D).

### 3.9. Differences in Cecal Microbial Community Structure

**Figure 3:**
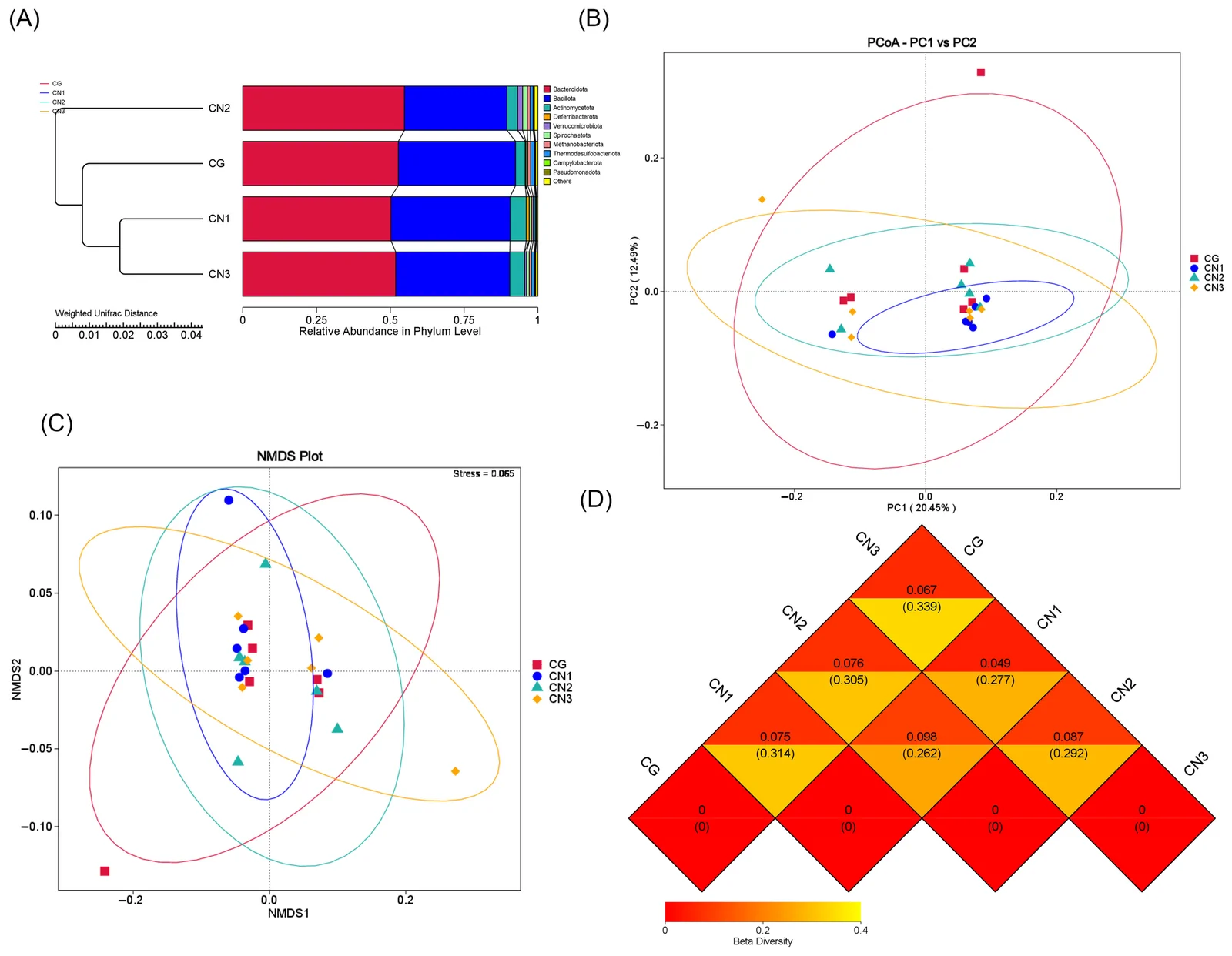
Beta diversity analysis based on unweighted UniFrac distances. (**A**) UPGMA clustering tree. (**B**) PCoA analysis. (**C**) NMDS analysis. (**D**) Unweighted UniFrac. Values in the matrix cells denote pairwise dissimilarity coefficients, with smaller values indicating greater similarity in community composition between sample pairs. For each cell, the top and bottom values represent Weighted UniFrac and Unweighted UniFrac distances, respectively. CG represented the control group, whereas CN1, CN2, and CN3 were experimental groups with dietary curcumin supplementation at 100, 200, and 400 mg/kg, respectively. Six samples were analyzed per group.

Beta diversity analysis was performed to assess the structural variations in cecal microbial communities across treatment groups. The unweighted pair-group method with arithmetic mean (UPGMA) clustering analysis was conducted based on microbial phylogenetic distances. Samples with the minimum pairwise distances were successively clustered to generate new nodes, and the average distances between newly formed nodes and remaining samples were calculated iteratively to construct a complete sample clustering tree (Figure 3A). Principal coordinates analysis (PCoA) was performed using unweighted UniFrac distances, where closer spatial distances between samples indicated higher similarity in microbial community composition (Figure 3B). Non-metric multi-dimensional scaling (NMDS) analysis yielded a stress value of 0.065, which was below the threshold of 0.2, confirming that the NMDS ordination reliably reflected the actual microbial dissimilarities among all samples (Figure 3C). In addition, a heat map based on the unweighted UniFrac distance matrix was generated to visualize the overall beta diversity profiles across samples (Figure 3D).

Further distance coefficient analysis revealed that the CN1 and CN3 groups exhibited the highest similarity in microbial community composition at the phylum level, with weighted and unweighted UniFrac distances of 0.049 and 0.277, respectively. For inter-group comparisons with the CG, the weighted and unweighted UniFrac distances were 0.057 and 0.314 (CG vs. CN1), 0.076 and 0.305 (CG vs. CN2), as well as 0.067 and 0.339 (CG vs. CN3), respectively.

### 3.10. Changes in Cecal Microbial Composition at Different Taxonomic Levels

**Figure 4:**
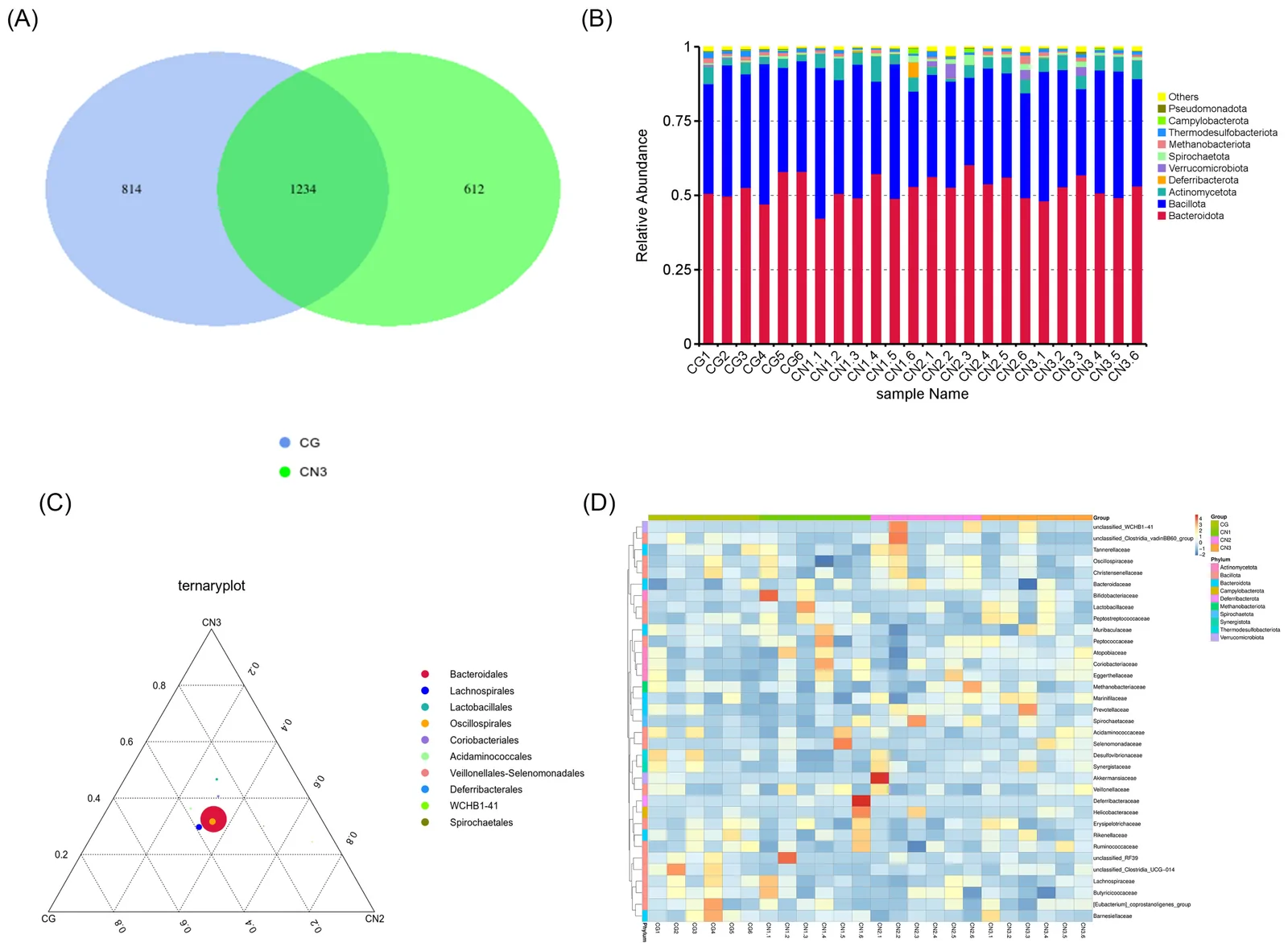
Microbial community composition at different taxonomic levels. (**A**) ASV Venn Diagram. (**B**) Phylum-level composition of the gut microbiota. (**C**) Order-level ternary plot. (**D**) Hierarchical Clustering Heatmap. CG represented the control group, whereas CN1, CN2, and CN3 were experimental groups with dietary curcumin supplementation at 100, 200, and 400 mg/kg, respectively. Six samples were analyzed per group.

Further profiling of cecal microbial relative abundance at multiple taxonomic levels confirmed that dietary curcumin supplementation effectively modulated the microbial community structure of aged laying hens. Venn analysis of amplicon sequence variants (ASVs) revealed distinct microbial compositional differences between the CG and CN3 groups, with 814 unique ASVs detected in the CG and 612 unique ASVs in the CN3 group (Figure 4A). At the phylum level, the top ten dominant bacterial phyla across all groups were identified as *Bacteroidota*, *Bacillota*, *Actinomycetota*, *Deferribacterota*, *Verrucomicrobiota*, *Spirochaetota*, *Methanobacteriota*, *Thermodesulfobacteriota*, *Campylobacterota*, and *Pseudomonadota* (Figure 4B). Ternary plot analysis at the order level showed that the nodes representing *Lactobacillales* were closely clustered toward the vertex of the CN3 group, indicating a higher relative abundance of *Lactobacillales* in the CN3 treatment (Figure 4C). Furthermore, the top 35 microbial taxa with the highest relative abundance at the family level were screened. Based on the abundance profiles of individual samples, hierarchical clustering analysis was performed, and the resulting abundance patterns were visualized in a heatmap (Figure 4D).

### 3.11. Analysis of Differentially Abundant Cecal Microbial Taxa

**Figure 5:**
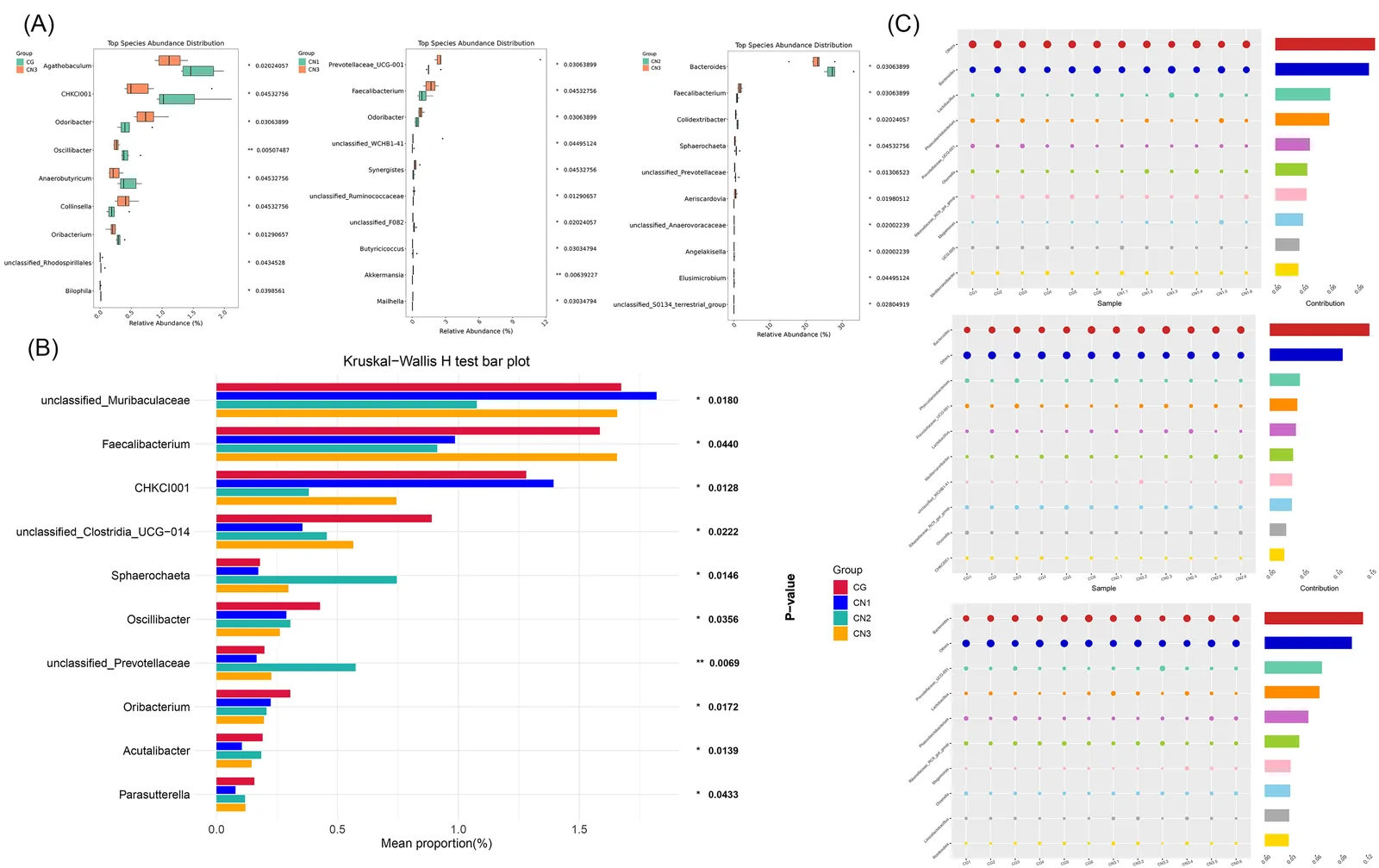
Differences in beneficial gut bacteria assessed using nonparametric tests and SIMPER analysis. (**A**) Wilcoxon rank sum test bar chart. (**B**) Kruskal–Wallis rank sum test bar chart. (**C**) Contributions of microbial taxa to community dissimilarity determined by SIMPER analysis. * *p* < 0.05, ** *p* < 0.01. CG represented the control group, whereas CN1, CN2, and CN3 were experimental groups with dietary curcumin supplementation at 100, 200, and 400 mg/kg, respectively. Six samples were analyzed per group.

The Wilcoxon rank-sum test for two independent samples was performed to screen microbial taxa with significant differences between groups (Figure 5A). Analysis of the top 10 microbial genera revealed distinct variations in key beneficial bacteria among treatments. At the genus level, the relative abundances of *Odoribacter* and *Collinsella* were significantly higher in the CN3 group than in the CG (*p* < 0.05). Compared with the CN1 group, the CN3 group exhibited markedly enriched *Faecalibacterium* and *Odoribacter* (*p* < 0.05). Additionally, the abundances of *Faecalibacterium* and *Bacteroides* were significantly elevated in the CN3 group relative to the CN2 group (*p* < 0.05). Furthermore, the Kruskal–Wallis H test indicated significant overall differences in the relative abundances of Faecalibacterium and Odoribacter among the four groups (*p* < 0.05), with the highest values observed in the CN3 group (Figure 5B). Similarity percentage (SIMPER) analysis was subsequently conducted to quantify the contribution of individual microbial taxa to the community dissimilarity between the control and curcumin supplementation groups, and the top 10 taxa contributing to inter-group differences were visualized according to their relative abundances (Figure 5C). Further analysis of differential beneficial bacteria showed that compared with the CG, the CN1 and CN2 groups were both enriched with *Lactobacillus* and *Phascolarctobacterium*. In contrast, the CN3 group exhibited significant enrichment of four beneficial genera, including *Faecalibacterium*, *Odoribacter*, *Lactobacillus* and *Phascolarctobacterium*, relative to the CG.

### 3.12. Phylogenetic Relationship Analysis of Dominant Microbial Genera

**Figure 6:**
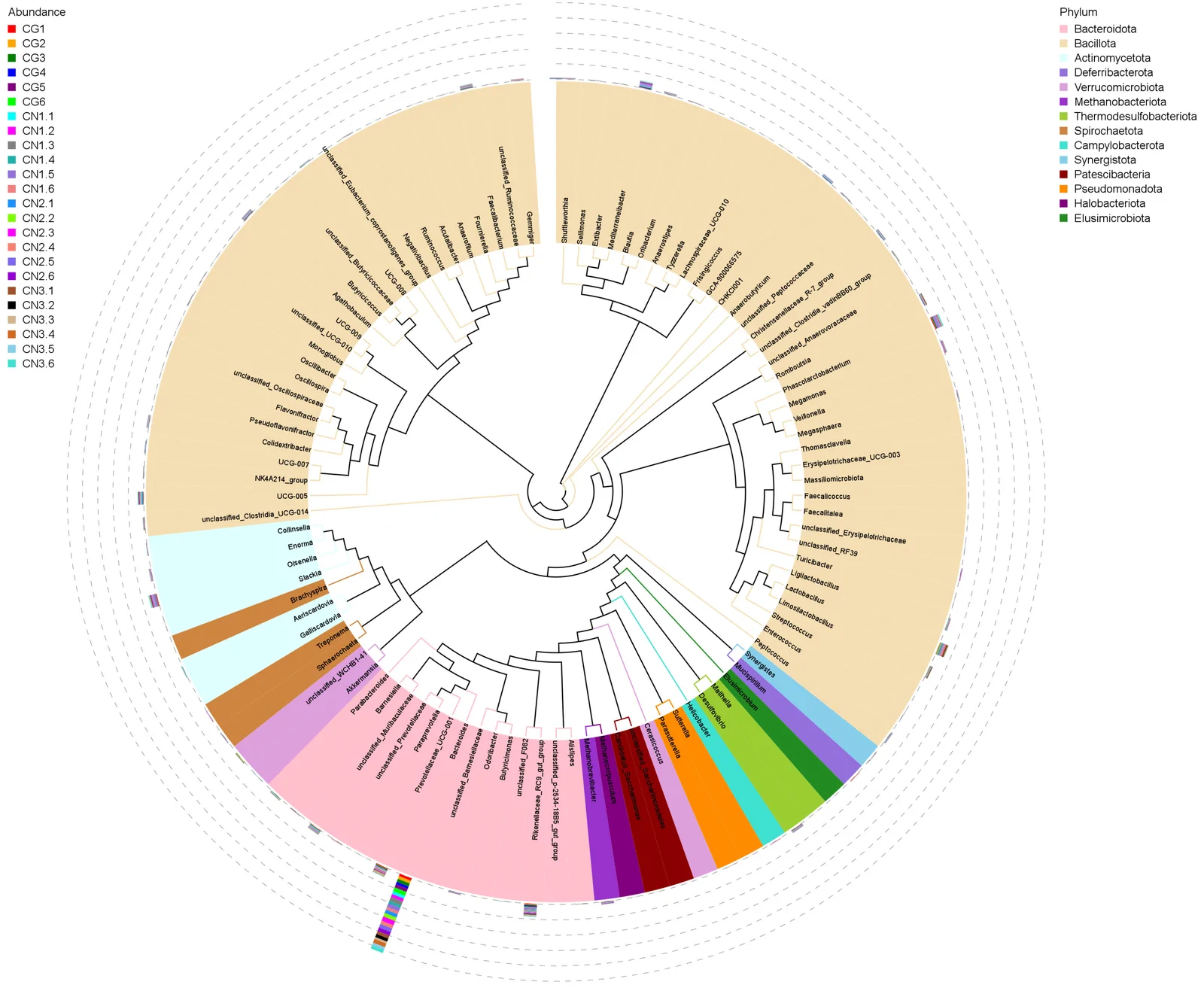
Circular evolutionary tree based on genus-level relative abundance reveals functional differentiation of cecal microbiota. CG represented the control group, whereas CN1, CN2, and CN3 were experimental groups with dietary curcumin supplementation at 100, 200, and 400 mg/kg, respectively. Six samples were analyzed per group.

To further explore the phylogenetic relationships of dominant microbial taxa at the genus level, representative sequences of the top 100 genera were obtained via multiple sequence alignment. A circular phylogenetic tree was constructed based on the relative abundances of these dominant genera, and the cecal microbial community was clustered into three distinct functional clusters (Figure 6). Cluster I (upper-left to upper-right sector, phylum *Bacillota*) was predominantly characterized by butyrate-producing genera including *Roseburia* and *Megamonas*, as well as lactic acid bacteria *Lactobacillus* and *Limosilactobacillus*, and this cluster was significantly enriched in the CN3 group. Cluster II (lower-left sector, phylum Spirochaetota) was mainly composed of fiber-fermenting bacteria *Treponema* and *Sphaerochaeta*, showing specific enrichment in the CN2 group. Cluster III (bottom region, phylum *Bacteroidota*) was dominated by *Prevotellaceae_UCG-001*, with higher abundance observed in the CN3 group. In addition, the relative abundances of potentially pathogenic genera Staphylococcus and *Bilophila* were markedly reduced in curcumin-supplemented groups. Specifically, the abundance of *Staphylococcus* decreased gradually with increasing dietary curcumin levels. Furthermore, all curcumin-treated groups exhibited significantly lower *Bilophila* abundance than the control group (*p* < 0.05) (Figure S1).

## 4. Discussion

Egg weight is a key economic trait that influences market value and profitability in laying hen production. Together with egg production rate, egg weight is regarded as one of the most critical indicators for evaluating laying performance, with a prominent contribution to total egg yield [17]. Previous studies have reported inconsistent effects of curcumin supplementation on laying performance parameters. Zou et al. [18] demonstrated that dietary curcumin significantly increased egg weight in 58-week-old Hy-Line Brown laying hens. In contrast, Liu et al. [19] found that curcumin supplementation improved egg production rate, feed conversion ratio, eggshell strength and eggshell thickness in 40-week-old peak-laying hens, whereas it exerted no significant influence on average egg weight. In the present study, the average egg weight was higher in CN3 than in CG, representing an increase of approximately 11.9%, whereas egg production rate and feed-to-egg ratio were not significantly affected. The pre-experimental average egg weight did not differ significantly among groups, and hens were stratified by baseline egg weight before random allocation, reducing the likelihood that baseline imbalance accounted for the observed treatment effect. Differences among studies may be related to hen age and breed, basal diet, curcumin dosage and bioavailability, and experimental duration. In particular, both the present study and that of Zou et al. [18] used late-phase laying hens, whereas Liu et al. [19] used peak-laying hens with a different physiological status. The increase in egg weight observed in CN3 may be partly associated with the improvements in intestinal morphology, tight-junction gene expression, antioxidant status, and inflammatory status, which could support nutrient utilization and the physiological processes involved in egg formation. In late-phase laying hens, potential modulation of hepatic lipid metabolism and yolk-precursor availability may also be relevant. However, nutrient digestibility, hepatic lipid metabolism, ovarian function, and egg-component weights were not measured in the present study; therefore, these mechanisms remain speculative. The relatively low average egg weight in CG may also have influenced the relative magnitude of the response. In addition, the small numerical difference between CN1 and CN2 (56.22 versus 55.74 g) should not be interpreted as evidence of a non-monotonic dose response, particularly given the absence of a significant quadratic effect. Therefore, the magnitude and underlying mechanisms of the egg-weight response should be interpreted cautiously and confirmed in longer-term studies incorporating longitudinal measurements and direct assessments of nutrient utilization and reproductive physiology.

Serum and intestinal oxidative biomarkers reliably reflect the dynamic balance between oxidative stress and antioxidant capacity in vivo. Antioxidant enzymes including SOD, GSH-Px, and CAT serve as core defensive components of the antioxidant system. Increased activities of these enzymes enhance free radical scavenging capacity and improve antioxidant status, whereas elevated MDA content, a final product of lipid peroxidation, indicates aggravated cellular oxidative damage [20]. Serum IgM, IgA, and IgY are major avian immunoglobulins, and increases in their concentrations generally suggest enhanced humoral immunity, while decreased levels imply immunosuppression or immune dysfunction [21]. Meanwhile, the balance among pro-inflammatory cytokines (IL-6 and TNF-α) and the anti-inflammatory/immunoregulatory cytokine IL-10 is indicative of immune regulation, and an altered balance among these mediators may reflect disturbed immune homeostasis rather than a uniform increase in inflammatory activity [22]. Multiple previous poultry studies have verified that dietary curcumin supplementation can effectively improve the antioxidant status of chickens, as evidenced by reported increases in serum SOD and CAT activity in those studies, upregulated antioxidant gene expression, and reduced MDA accumulation [23,24,25]. These reported findings are highly consistent with our current results. In this study, birds in the CN3 group had significantly higher serum CAT concentration and lower MDA content than those in the CG, with clear linear changes in antioxidant indicators as dietary curcumin levels increased. A similar antioxidant-modulating effect of curcumin was also documented in 240-day-old aged laying quails, where 200 mg/kg dietary curcumin reduced mortality, increased GSH-Px activity, and decreased serum MDA levels [26]. In addition to serum indicators, the present study also detected improved jejunal antioxidant profiles, including elevated GSH-Px and CAT concentrations and decreased MDA concentration, which further supports the antioxidant effects of curcumin in aged laying hens.

Apart from antioxidant modulation, curcumin also exerted positive regulatory effects on host immune and inflammatory responses in aged laying hens. In this study, serum and jejunal IgM, IgA, and IgY levels were significantly higher in the CN3 group than in the CG, displaying a distinct linear upward trend with increasing curcumin dosage. Regarding inflammatory mediators, the levels of IL-6 and TNF-α, which are primarily pro-inflammatory cytokines, were markedly decreased in the CN3 group. IL-10, an anti-inflammatory and immunoregulatory cytokine, was also reduced. This concurrent decrease in IL-10 should not be interpreted simply as a uniform reduction in inflammatory activity. Late-phase laying hens are in a physiologically aged stage characterized by chronic low-grade inflammation and compromised immune regulation [2,4,5]. Under such conditions, IL-10 is often upregulated as a negative feedback mechanism to counterbalance inflammatory stimulation. Therefore, the decrease in IL-10 following curcumin supplementation may reflect a reduced demand for compensatory anti-inflammatory signaling after the alleviation of inflammatory stress, or a shift toward restored immune regulatory balance, rather than a decline in anti-inflammatory capacity. The improved humoral immune function observed in curcumin-treated groups is consistent with a previous broiler study, which reported that 300–400 mg/kg curcumin nanoparticles produced the greatest increases in serum IgM, IgY, and IgA levels and enhanced host humoral immunity [27]. Moreover, previous studies have confirmed that curcumin supplementation inhibits the expression of pro-inflammatory cytokines such as IL-6, IL-1β, and TNF-α in early-stage laying hens and reduces circulating TNF-α, IL-2, and IL-6 levels in broilers [11], which supports the anti-inflammatory effect of curcumin observed in the current trial. In summary, dietary curcumin supplementation can effectively enhance systemic and intestinal antioxidant defense capacity, activate humoral immune responses, and attenuate pro-inflammatory stress in aged laying hens. Notably, the tested curcumin doses (100, 200, and 400 mg/kg) yielded consistent linear improvements in antioxidant, immune, and cytokine parameters, demonstrating a reliable dose-dependent regulatory effect of curcumin on the physiological health of late-phase laying hens.

Structural damage to the intestinal physical barrier is primarily manifested as reduced villus height and increased crypt depth. These morphological alterations lower the villus height/crypt depth (V/C) ratio and impair intestinal nutrient absorption efficiency. Intestinal barrier dysfunction also leads to increased intestinal permeability, which is closely associated with the downregulated mRNA expression of tight junction protein genes [28,29]. In this study, dietary curcumin supplementation markedly improved intestinal morphological characteristics in aged laying hens. However, the optimal dose varied among intestinal segments. In the duodenum and ileum, CN3 (400 mg/kg) showed the greatest improvements in villus height and V/C ratio, and the lowest crypt depth. In contrast, in the jejunum, CN2 (200 mg/kg) exhibited the lowest crypt depth and the highest V/C ratio, with significant quadratic effects (Table 8). Furthermore, the transcriptional levels of tight junction genes were significantly upregulated in curcumin-treated groups in a clear linear dose-dependent manner (Table 9). These results are strongly supported by previous poultry studies. Consistent with our findings, curcumin supplementation has been shown to optimize jejunal morphology, elevate the V/C ratio, reduce crypt depth, and upregulate intestinal OCLN mRNA expression in yellow-feathered broilers [30] and Ross 308 broilers [31]. Collectively, these observations demonstrate that curcumin strengthens intestinal physical barrier integrity by ameliorating intestinal structural damage and facilitating tight junction gene expression, which is crucial for maintaining intestinal homeostasis in aged laying hens. Nevertheless, the dose–response pattern was parameter-specific, and 400 mg/kg was not the optimal dose for every intestinal parameter, particularly jejunal crypt depth and V/C ratio.

To further clarify the potential mechanisms by which curcumin may improve intestinal morphological structure and barrier function in aged laying hens, 16S rRNA gene sequencing was performed to characterize alterations in cecal microbial community structure. Significant differences in alpha and beta diversity were detected between the control and curcumin supplementation groups. Following rank-based pairwise comparisons with Benjamini–Hochberg adjustment, significant differences in alpha diversity were observed between CG and CN1, between CG and CN3, and between CN1 and CN2, whereas the differences between CG and CN2 were not statistically significant. These findings indicate that curcumin supplementation was associated with alterations in cecal microbial diversity, although the response varied among supplementation levels and did not follow a uniform dose-dependent pattern. These compositional variations indicate that dietary curcumin was associated with remodeling of the intestinal microecological structure of aged laying hens [32]. Differential abundance analysis identified key microbial taxa that responded to curcumin supplementation. In the chicken cecum, *Lactobacillales* [33], *Faecalibacterium* [34], *Odoribacter* [35], *Roseburia* [36], and *Phascolarctobacterium* [37] are taxa known to include butyrate-producing members, whereas *Staphylococcus* [38] and *Bilophila* [39] include taxa that have been recognized as potential pathogens. In this study, the CN3 group exhibited the most distinct and favorable microbial characteristics, with significant enrichment of potential butyrate-producing taxa and pronounced depletion of *Staphylococcus* and *Bilophila*. These results suggest that an appropriate curcumin dose exerts a bidirectional regulatory effect on intestinal microflora, promoting the proliferation of potentially beneficial bacteria while suppressing the colonization of potentially harmful microbes.

Butyrate acts as the primary energy substrate for intestinal epithelial cells and participates in regulating epithelial cell proliferation and differentiation [40]. A previous study on aged Hy-Line Brown laying hens reported that exogenous butyrate supplementation significantly increased ileal villus height and upregulated the mRNA levels of intestinal tight junction genes (*OCLN* and *CLDN1*) [41]. This finding is consistent with, and may help explain, the improved intestinal morphology observed in curcumin-treated groups, especially the improved intestinal structure in the CN3 group. The increased abundance of butyrate-producing microbes in the CN3 group may be associated with increased intestinal butyrate production, which could potentially contribute to intestinal epithelial cell growth, villus development, and reduced crypt depth. However, intestinal butyrate concentration was not directly measured in the present study, and this proposed mechanism therefore remains speculative. To date, few studies have explored the effects of curcumin supplementation on intestinal microflora in aged laying hens. Nevertheless, the ability of curcumin to modulate the microbiota has been reported in diverse animal models, including growing laying hens [11], broilers [42], ducks [43], mice [44], and primates [45]. Collectively, these studies suggest that curcumin can optimize intestinal microflora composition, enrich beneficial bacteria, and suppress pathogenic proliferation to maintain intestinal microecological homeostasis. In line with these cross-species findings, the present study further suggests that curcumin-induced intestinal morphological improvement in aged laying hens may be partly associated with the remodeled microbial community structure and potentially enhanced butyrate-producing functional capacity of cecal microbiota. Future studies with direct measurements of butyrate and causal approaches such as germ-free models or fecal microbiota transplantation are needed to validate this hypothesis.

Intestinal health, microbial homeostasis, and physiological and biochemical characteristics are synergistically interconnected rather than independently regulated in poultry [46,47]. Optimized intestinal morphology preserves physical barrier integrity and sustains immune homeostasis, which is critical for moderating systemic inflammatory responses [48]. Increased villus height and reduced crypt depth reflect improved epithelial maturation and mucosal health status [49]. The concurrent upregulation of tight junction protein mRNAs further diminishes intestinal permeability and blocks the translocation of luminal lipopolysaccharide (LPS) into the circulation [50]. As a pivotal pro-inflammatory stimulus, translocated LPS triggers hepatic Kupffer cell activation and initiates systemic inflammatory cascades [51]. In the present study, curcumin supplementation markedly decreased serum and jejunal IL-6 and TNF-α levels, concurrent with improved intestinal morphology and enhanced tight junction protein mRNA expression. These findings are consistent with the proposed barrier–LPS–inflammation pathway; however, intestinal permeability and circulating LPS concentrations were not directly measured. Therefore, this pathway should be regarded as a potential mechanism rather than an experimentally demonstrated causal relationship, and future studies are needed to directly test it. These findings support the possibility of a continuous regulatory pathway whereby curcumin strengthens intestinal barrier function, restrains LPS translocation, and consequently mitigates systemic inflammation in aged laying hens. Moreover, alterations in cecal microbial community structure were closely associated with host antioxidant capacity. The CN3 group exhibited superior antioxidant profiles, as evidenced by elevated jejunal GSH-Px and CAT concentrations, reduced jejunal MDA accumulation, and improved serum CAT and MDA status. These favorable antioxidant alterations corresponded well with the pronounced enrichment of potential butyrate-producing microbes in the CN3 group. Existing mechanistic studies have shown that butyrate can act as a key functional metabolite mediating antioxidant and anti-inflammatory activities. Specifically, butyrate has been reported to activate the *Nrf2–Keap1* signaling pathway to upregulate the transcription and activity of antioxidant enzymes including SOD, CAT, and GSH-Px. Meanwhile, it has been shown to suppress excessive activation of the *NF-κB* signaling cascade, thereby inhibiting the production of pro-inflammatory IL-6 and TNF-α [52,53].

Building on the above phenotypic findings and proposed mechanisms, the present study systematically explores the potential protective effects and possible mechanisms of curcumin against intestinal health deterioration in aged laying hens. This study combined 16S rRNA gene sequencing of the microbiota, assessment of intestinal morphology, measurement of serum and jejunal oxidative stress and inflammatory markers, and quantification of tight junction gene expression. Hens in the late laying period frequently undergo declining hormonal function and persistent metabolic stress, which collectively trigger intestinal barrier damage, excessive oxidative stress, and microbial dysbiosis. By integrating microbial composition changes, mucosal structural improvement, tight junction gene expression changes, and host physiological responses, the current findings provide a basis for the application of curcumin as a promising functional regulator to maintain intestinal health in aged laying hens and support its potential use in the health management of aging poultry. Despite these comprehensive observations, several avenues warrant further investigation. Although we observed significant microbial shifts, the functional metabolic network linking curcumin intervention to host physiology remains obscure. Future integration of metatranscriptomics and metabolomics is required to clarify the interactions among curcumin, gut microbiota, and their functional metabolites. The causal relationship between the enrichment of butyrate-producing genera and intestinal barrier improvement should be validated using germ-free chicken models or fecal microbiota transplantation (FMT) assays. To translate these findings into commercial practice, large-scale, multi-site dose–response trials are needed to determine the optimal inclusion rate and intervention timing for different laying stages, thereby establishing a standardized application protocol for the poultry industry.

## 5. Conclusions

**Figure 7:**
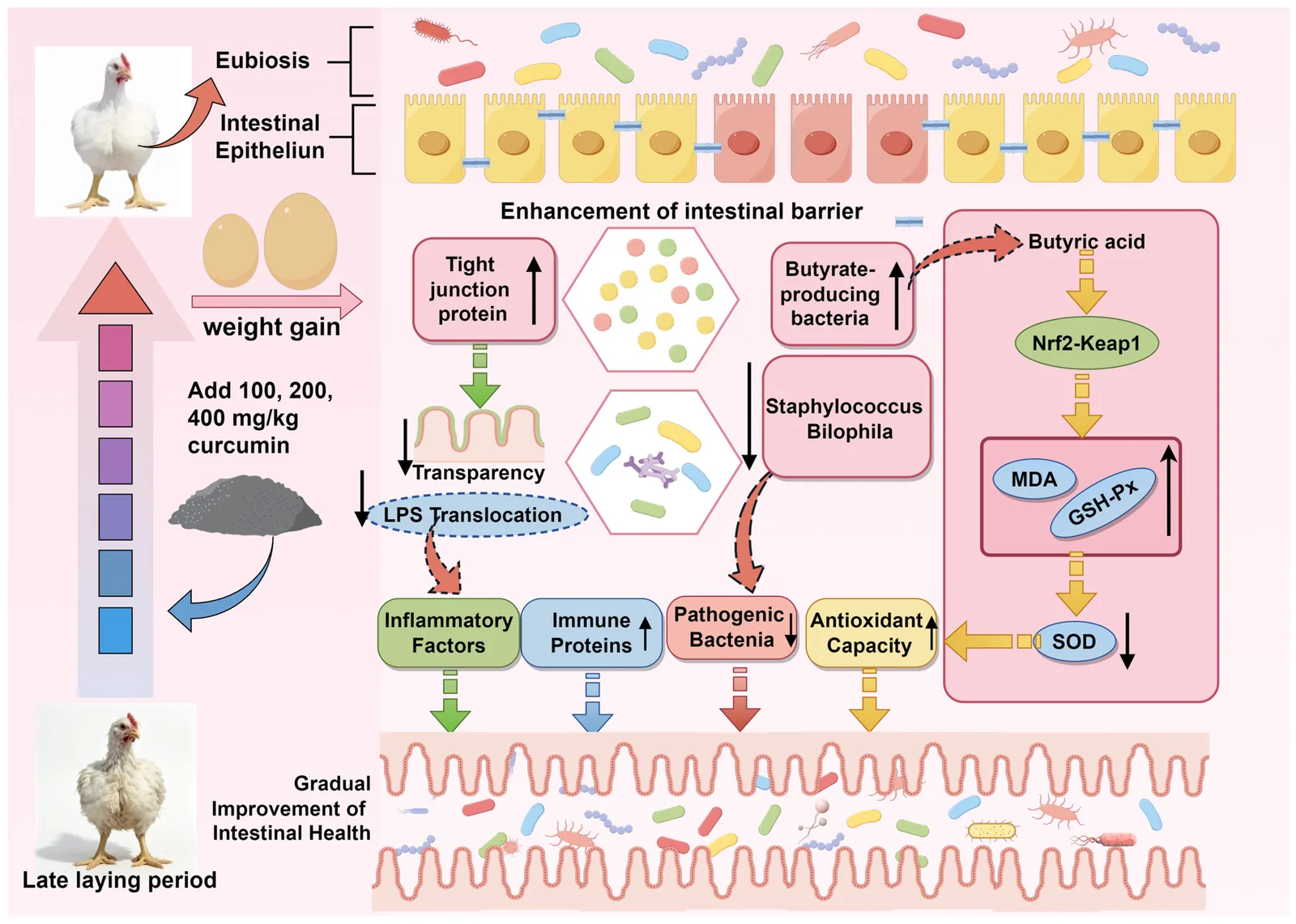
Proposed mechanisms underlying the effects of dietary curcumin in late-laying hens. Solid arrows indicate responses experimentally measured in the present study; dashed arrows indicate hypothesized pathways that require further validation. Microbial changes were associated with, but not experimentally proven to mediate, improvements in intestinal barrier function, antioxidant status, or immune responses.

In conclusion, graded dietary curcumin supplementation effectively remodeled cecal microbial composition, optimized intestinal morphology and barrier integrity, and enhanced antioxidant capacity and immune homeostasis in aged laying hens. Among the tested doses, 400 mg/kg curcumin produced the most consistent overall responses. Specifically, this dose markedly increased villus height, reduced crypt depth, and elevated the villus height/crypt depth ratio, accompanied by significant upregulation of intestinal tight junction gene expression. Microbial profiling further verified that curcumin supplementation increased the relative abundance of key beneficial butyrate-producing taxa, including *Lactobacillales*, *Odoribacter*, *Roseburia*, *Faecalibacterium*, and *Phascolarctobacterium*, while distinctly inhibiting the colonization of potentially pathogenic genera *Staphylococcus* and *Bilophila*. In addition, curcumin intervention improved both systemic and intestinal antioxidant profiles. Increased levels of IgA, IgY, and IgM in serum and jejunal mucosa, together with reduced pro-inflammatory cytokine accumulation, effectively strengthened host immune defense function. Collectively, these findings demonstrate that curcumin can serve as an effective functional feed additive for aged laying hens. It ameliorates intestinal structural damage, improves nutrient absorption efficiency, and enhances antioxidant and immune defenses, thereby comprehensively improving the health status of hens during the late laying period. Among the tested doses, 400 mg/kg produced the most consistent overall responses; however, because higher doses were not evaluated, the true optimal inclusion rate remains to be determined in future dose–response studies. The proposed mechanisms are illustrated in Figure 7.

## Figures and Tables

**Table 1:** Composition and nutrient levels of the basal diet for late-phase laying hens (kg/100 kg diet).

Ingredients	Contents	Nutrient Components	Contents
Corn	59.50	Metabolizable energy ^2^, MJ/kg	11.90
Soybean meal	20.50	Crude protein ^3^	14.50
Soybean oil	1.20	Calcium ^3^	4.20
Calcium hydrogen phosphate	0.60	Available phosphorus ^3^	0.30
Salt	0.25	Lysine ^2^	0.72
Methionine	0.06	Methionine ^2^	0.32
Lysine (98%)	0.05	Cystine ^2^	0.28
Threonine	0.01	Threonine ^2^	0.58
Limestone	9.50		
Oyster shell powder	1.50		
Fish meal	1.50		
Wheat bran	5.00		
Premix ^1^	1.00		

^1^ The premix provides per kilogram of diet: VA 4000 IU, VD_3_ 3500 IU, VE 50 mg, VK 3.0 mg, VB_1_ 2.5 mg, VB_2_ 8.0 mg, VB_6_ 5.0 mg, VB_12_ 0.03 mg, folic acid 50 mg, niacin 50 mg, pantothenic acid 15 mg, biotin 0.30 mg, choline chloride 500 mg, Se as Na_2_SeO_3_ 0.30 mg, I as KIO_3_ 0.35 mg, Cu as CuSO_4_·5H_2_O 8 mg, Mn as MnSO_4_·H_2_O 50 mg, Fe as FeSO_4_·7H_2_O 60 mg, Zn as ZnSO_4_·7H_2_O 60 mg. ^2^ Calculated values. ^3^ Analyzed values.

**Table 2:** Nucleotide sequences of specific primers.

Genes	Primer Sequence (5′-3′)	Product Length (bp)	Accession Number
*ZO-1*	F: CAGATGGCCATAGGGGTGAC	105	XM_040706827
	R: ACAGGTAGCACCATTGGGAT		
*OCLN*	F: CTGCCATTTTAAGGGCCACC	147	XM_025144247
	R: GAGCGTCGTCCACGTAG		
*CLDN1*	F: AACGGCTAGCAAACCCCAA	106	NM_001013611
	R: CAATGCAGCCCCCTAGAGA		
*Act-β*	F: CATTGTCCACCGCAAATGCT	108	NM_205518
	R: AGCCATGCCAATCTCGTCTT		
*GAPDH*	F: GGGGAAGTCATCCCTGAGC	111	NM_204305
	R: TTGGCTGTGTGTTTCCAGAGG		

*ZO-1*, *Zonula Occludens-1*; *OCLN*, *occludin*; *CLDN1*, *claudin 1*; *Act-β, Actin-β*; *GAPDH*, *Glyceraldehyde-3-phosphate Dehydrogenase*.

**Table 3:** Effects of curcumin supplementation on production performance of late-laying hens.

Items	Groups				SEM	*p*-Value		
	CG	CN1	CN2	CN3		Anova	Linear	Quadratic
ADFI, g/d	113.25	114.67	111.58	123.17	4.94	0.847	0.576	0.578
EPR, %	62.11	56.38	59.24	63.24	2.47	0.785	0.789	0.356
AEW, g	52.17 ^b^	56.22 ^ab^	55.74 ^ab^	58.35 ^a^	1.35	0.031	0.008	0.364
FER	3.48	3.60	3.36	3.35	0.23	0.842	0.556	0.907

ADFI, Average daily feed intake; EPR, Egg production rate; AEW: Average egg weight; FER: Feed-to-egg ratio. CG, Control group; CN1, Basal diet + 100 mg/kg curcumin; CN2, Basal diet + 200 mg/kg curcumin; CN3, Basal diet + 400 mg/kg curcumin; SEM, standard error of the mean. ^a,b^ Within the same row, values with different lowercase letter superscripts denote significant differences (*p* < 0.05).

**Table 4:** Effects of curcumin supplementation on serum antioxidant indices of late-laying hens.

Items	Groups				SEM	*p*-Value		
	CG	CN1	CN2	CN3		Anova	Linear	Quadratic
GSH-Px, pmol/mL	11.85	12.23	12.83	13.62	0.45	0.555	0.163	0.824
SOD, pg/mL	20.86	22.04	24.91	28.02	1.72	0.486	0.134	0.785
MDA, pg/mL	362.53 ^a^	311.61 ^ac^	263.29 ^bc^	242.32 ^b^	14.22	0.005	<0.0001	0.501
CAT, ng/L	41.79 ^c^	49.08 ^bc^	56.30 ^b^	78.64 ^a^	3.49	<0.0001	<0.0001	0.092

GSH-Px, Glutathione Peroxidase; SOD, Superoxide Dismutase; MDA, Malondialdehyde; CAT, Catalase; CG, Control group; CN1, Basal diet + 100 mg/kg curcumin; CN2, Basal diet + 200 mg/kg curcumin; CN3, Basal diet + 400 mg/kg curcumin; SEM, standard error of the mean. ^a,b,c^ Within the same row, values with different lowercase letter superscripts denote significant differences (*p* < 0.05).

**Table 5:** Effects of curcumin supplementation on serum inflammatory and immune indices of late-laying hens.

Items	Groups				SEM	*p*-Value		
	CG	CN1	CN2	CN3		Anova	Linear	Quadratic
IgM, ng/mL	4696 ^b^	4846 ^b^	4991 ^b^	6004 ^a^	176	0.025	0.007	0.167
IgA, ng/mL	6034 ^c^	6333 ^bc^	8667 ^a^	6933 ^b^	245	<0.0001	<0.0001	<0.0001
IgY, μg/mL	62.36 ^b^	69.78 ^ab^	77.32 ^ab^	84.53 ^a^	2.94	0.034	0.004	0.983
IL-10, ng/L	135.45 ^a^	109.68 ^ab^	104.90 ^ab^	84.65 ^b^	6.32	0.031	0.004	0.817
IL-6, ng/L	32.71 ^a^	30.69 ^a^	30.86 ^a^	19.97 ^b^	1.78	0.033	0.012	0.164
TNF-α, ng/L	127.22 ^a^	124.34 ^ac^	108.98 ^bc^	103.41 ^b^	3.41	0.018	0.003	0.917

IgM, Immunoglobulin M; IgA, Immunoglobulin A; IgY, Immunoglobulin Y; IL-10, Interleukin-10; IL-6, Interleukin-6; TNF-α, Tumor Necrosis Factor-alpha; CG, Control group; CN1, Basal diet + 100 mg/kg curcumin; CN2, Basal diet + 200 mg/kg curcumin; CN3, Basal diet + 400 mg/kg curcumin; SEM, standard error of the mean. ^a,b,c^ Within the same row, values with different lowercase letter superscripts denote significant differences (*p* < 0.05).

**Table 6:** Effects of curcumin supplementation on jejunal antioxidant indices of late-laying hens.

Items	Groups				SEM	*p*-Value		
	CG	CN1	CN2	CN3		Anova	Linear	Quadratic
GSH-Px, pmol/mg protein	16.09 ^c^	18.48 ^b^	20.56 ^b^	23.39 ^a^	0.71	<0.0001	<0.0001	0.816
SOD, pg/mg protein	18.36	20.00	21.63	23.28	1.01	0.366	0.082	0.996
MDA, pg/mg protein	658.18 ^a^	567.01 ^ab^	478.77 ^bc^	398.98 ^c^	25.67	<0.0001	<0.0001	0.876
CAT, ng/mg protein	67.05 ^c^	77.93 ^b^	84.21 ^ab^	90.20 ^a^	2.11	<0.0001	<0.0001	0.318

GSH-Px, Glutathione Peroxidase; SOD, Superoxide Dismutase; MDA, Malondialdehyde; CAT, Catalase; CG, Control group; CN1, Basal diet + 100 mg/kg curcumin; CN2, Basal diet + 200 mg/kg curcumin; CN3, Basal diet + 400 mg/kg curcumin; SEM, standard error of the mean. ^a,b,c^ Within the same row, values with different lowercase letter superscripts denote significant differences (*p* < 0.05).

**Table 7:** Effects of curcumin supplementation on jejunal inflammatory and immune indices of late-laying hens.

Items	Groups				SEM	*p*-Value		
	CG	CN1	CN2	CN3		Anova	Linear	Quadratic
IgM, ng/mg protein	3109 ^c^	3569 ^bc^	3924 ^ab^	4172 ^a^	120	0.004	<0.0001	0.577
IgA, ng/mg protein	4017 ^b^	4342 ^ab^	4673 ^ab^	5013 ^a^	134	0.040	0.005	0.976
IgY, μg/mg protein	90.05 ^b^	129.14 ^a^	136.57 ^a^	139.40 ^a^	6.10	0.006	0.002	0.069
IL-10, ng/mg protein	319.22 ^a^	253.83 ^bc^	260.32 ^b^	201.56 ^c^	12.39	0.003	<0.0001	0.864
IL-6, ng/mg protein	107.30	110.35	87.56	106.41	4.46	0.259	0.520	0.398
TNF-α, ng/mg protein	404.72 ^a^	317.68 ^ab^	305.39 ^ab^	236.58 ^b^	21.82	0.045	0.007	0.819

IgM, Immunoglobulin M; IgA, Immunoglobulin A; IgY, Immunoglobulin Y; IL-10, Interleukin-10; IL-6, Interleukin-6; TNF-α, Tumor Necrosis Factor-alpha; CG, Control group; CN1, Basal diet + 100 mg/kg curcumin; CN2, Basal diet + 200 mg/kg curcumin; CN3, Basal diet + 400 mg/kg curcumin; SEM: standard error of the mean. ^a,b,c^ Within the same row, values with different lowercase letter superscripts denote significant differences (*p* < 0.05).

**Table 8:** Effects of curcumin supplementation on intestinal morphology of late-laying hens.

Items	Groups				SEM	*p*-Value		
	CG	CN1	CN2	CN3		Anova	Linear	Quadratic
Duodenum								
Villus height (µm)	690.04 ^c^	744.75 ^c^	769.44 ^b^	817.68 ^ab^	14.20	0.006	<0.0001	0.888
Crypt depth (µm)	126.61 ^a^	130.50 ^a^	112.74 ^ab^	106.80 ^b^	3.53	0.042	0.011	0.427
V/C	5.55 ^c^	5.75 ^bc^	6.85 ^ab^	7.91 ^a^	0.27	0.002	<0.0001	0.305
Jejunum								
Villus height (µm)	696.25 ^b^	722.43 ^ab^	729.90 ^ab^	766.11 ^a^	9.25	0.048	0.007	0.752
Crypt depth (µm)	126.01 ^a^	122.46 ^a^	95.85 ^b^	117.21 ^a^	3.02	<0.0001	0.005	0.004
V/C	5.65	5.94	7.68	6.60	0.22	0.001	0.005	0.048
Ileum								
Villus height (µm)	597.55 ^b^	611.30 ^bc^	651.26 ^a^	644.45 ^ac^	7.15	0.010	0.002	0.392
Crypt depth (µm)	127.09 ^a^	123.48 ^a^	110.37 ^b^	112.85 ^b^	1.99	<0.0001	<0.0001	0.220
V/C	4.69 ^b^	4.95 ^b^	5.94 ^a^	5.74 ^a^	0.14	<0.0001	<0.0001	0.257

V/C, Villus height/Crypt depth; CG, Control group; CN1, Basal diet + 100 mg/kg curcumin; CN2, Basal diet + 200 mg/kg curcumin; CN3, Basal diet + 400 mg/kg curcumin; SEM, standard error of the mean. ^a,b,c^ Within the same row, values with different lowercase letter superscripts denote significant differences (*p* < 0.05).

**Table 9:** Effects of curcumin supplementation on intestinal tight junction protein mRNA expression of late-laying hens.

Items	Groups				SEM	*p*-Value		
	CG	CN1	CN2	CN3		Anova	Linear	Quadratic
Duodenum								
*ZO-1*	1 ^b^	1.50 ^ab^	1.66 ^a^	1.85 ^a^	0.10	0.016	0.002	0.379
*Occludin*	1 ^c^	1.54 ^b^	1.70 ^ab^	1.95 ^a^	0.09	<0.0001	<0.0001	0.228
*Claudin-1*	1 ^c^	1.23 ^b^	1.08 ^a^	1.15 ^a^	0.02	<0.0001	0.016	0.006
Jejunum								
*ZO-1*	1 ^b^	1.42 ^ab^	1.59 ^a^	1.71 ^a^	0.09	0.026	0.004	0.356
*Occludin*	1 ^d^	1.63 ^c^	2.04 ^a^	1.79 ^b^	0.08	<0.0001	<0.0001	<0.0001
*Claudin-1*	1 ^b^	1.70 ^a^	1.90 ^a^	1.94 ^a^	0.10	<0.0001	<0.0001	0.020
Ileum								
*ZO-1*	1 ^d^	1.58 ^c^	1.78 ^b^	1.93 ^a^	0.08	<0.001	<0.001	<0.001
*Occludin*	1 ^c^	1.54 ^bc^	1.71 ^b^	1.84 ^a^	0.07	<0.001	<0.001	<0.001
*Claudin-1*	1 ^d^	1.33 ^c^	1.77 ^b^	2.70 ^a^	0.16	<0.001	<0.001	0.122

CG, Control group; CN1, Basal diet + 100 mg/kg curcumin; CN2, Basal diet + 200 mg/kg curcumin; CN3, Basal diet + 400 mg/kg curcumin; SEM, standard error of the mean. ^a,b,c,d^ Within the same row, values with different lowercase letter superscripts denote significant differences (*p* < 0.05).

## Data Availability

The sequencing data for each sample are publicly available in the NCBI Sequence Read Archive under BioProject accession number PRJNA1468589.

## Supplementary Materials

The following supporting information can be downloaded at: https://www.mdpi.com/article/10.3390/ani16213415/s1, Figure S1: Pathogen grading chart; Table S1. Baseline production performance of Jingfen No. 1 laying hens at 60 weeks of age before curcumin supplementation; Table S2. Weekly production performance of late-laying hens supplemented with graded levels of curcumin.

## References

[1] Liu J., Fu Y., Zhou S., Zhao P., Zhao J., Yang Q., Wu H., Ding M., Li Y. (2023). Comparison of the effect of quercetin and daidzein on production performance, anti-oxidation, hormones, and cecal microflora in laying hens during the late laying period. Poult. Sci..

[2] Nii T., Sugiura T., Suzuki N., Isobe N., Yoshimura Y. (2025). Effects of Aging on the Microbiota and Inflammatory Status of the Intestinal and Oviductal Mucosa in Laying Hens. Poult. Sci..

[3] Shi K., Liu X., Duan Y., Jiang X., Li N., Du Y., Li D., Feng C. (2024). Dynamic Changes in Intestinal Gene Expression and Microbiota across Chicken Egg-Laying Stages. Animals.

[4] Kuroda A., Jaisue J., Isobe N., Nii T. (2026). Age-related changes in intestinal mucosal barrier function in laying hens during the mid-to-late production stages. Poult. Sci..

[5] Hu Z., Wu L., Lv Y., Ge C., Luo X., Zhan S., Huang W., Shen X., Yu D., Liu B. (2025). Integrated analysis of microbiome and transcriptome reveals the mechanisms underlying the chlorogenic acid-mediated attenuation of oxidative stress and systemic inflamm tory responses via gut-liver axis in post-peaking laying hens. J. Anim. Sci. Biotechnol..

[6] Huang Z., Wang G., Xu M., Shi Y., Feng J., Zhang M., Li C. (2025). Puerarin Enhances Eggshell Quality by Mitigating Uterine Senescence in Late-Phase Laying Breeder Hens. Antioxidants.

[7] Amevor F.K., Cui Z., Du X., Ning Z., Deng X., Xu D., Shu G., Wu Y., Cao X., Shuo W. (2022). Supplementation of Dietary Quercetin and Vitamin E Promotes the Intestinal Structure and Immune Barrier Integrity in Aged Breeder Hens. Front. Immunol..

[8] Guo Y., Xu Y., Wang D., Yang S., Song Z., Li R., He X. (2024). Dietary silymarin improves performance by altering hepatic lipid metabolism and cecal microbiota function and its metabolites in late laying hens. J. Anim. Sci. Biotechnol..

[9] Tang X., Zhang B., Yang J., Xie Y., Xiong K. (2026). Curcumin as a Green Antibiotic Substitute: Mechanisms and Applications in Poultry Production and Health Promotion. Animals.

[10] Yang Q., Cheng L., Zhang T., Yaron S., Jiang H., Sui Z., Corke H. (2020). Phenolic profiles, antioxidant, and antiproliferative activities of turmeric (*Curcuma longa*). Ind. Crops Prod..

[11] Xu Z., Zhu W., Xu D., Amevor F.K., Wu Y., Ma D., Cao X., Wei S., Shu G., Zhao X. (2024). Supplementation of curcumin promotes the intestinal structure, immune barrier function and cecal microbiota composition of laying hens in early laying period. Poult. Sci..

[12] Xu Y., Huang Y., Zhu Y., Wang J., Elsenousey H.K., Fouad A.M., Lin X., Zhang S., Han Y., Yan S. (2026). Maternal curcumin supplementation alleviates intestinal inflammation of Escherichia coli-infected offspring via modulating gut microbiome in chickens. Anim. Nutr..

[13] Zhang H.-D., Zhao X.-F., Ren Z.-Z., Tong M.-Q., Chen J.-N., Li S.-Y., Chen H., Wang D.-H. (2021). Comparison between different breeds of laying hens in terms of eggshell translucency and its distribution in various ends of the eggshell. Poult. Sci..

[14] Nawab A., Li G., Liu W., Lan R., Wu J., Zhao Y., Kang K., Kieser B., Sun C., Tang S. (2019). Effect of dietary curcumin on the antioxidant status of laying hens under high-temperature condition. J. Therm. Biol..

[15] Wu H., Chai H., Yu X., Zhao D., Liu H., Peng W., Ji F., Xu L., Hou G. (2026). Alpinia katsumadai and Wurfbainia vera Extracts Modulate Antioxidant Function and Intestinal Morphology in Danzhou Chickens via Gut Microbiota–Metabolite Interactions Involving Hydroxyoctadecadienoic Acid Metabolism and Bacteroidota Remodeling. Microorganisms.

[16] Yu X., Cao T., Ipemba E., Bakala G.B., Leveut L.G.D., Peng W., Ji F., Li H., Xu L., Wu H. (2025). Effects of dietary supplementation with bile acids on growth performance, antioxidant capacity, lipid metabolism, and cecal microbiota of Danzhou chickens. Poult. Sci..

[17] Ghaly M.M., El-Husseiny O. (2021). Best-fitted regression models for profitability of two egg-type commercial pullets. Trop. Anim. Health Prod..

[18] Zou C., Jiang H., Wu X., Gao J., Ma W. (2025). Integrative analysis of metabolomics and transcriptomics reveals alterations in egg quality and hepatic lipid metabolism in hens supplemented with curcumin. Anim. Nutr..

[19] Liu M., Lu Y., Gao P., Xie X., Li D., Yu D., Yu M. (2020). Effect of curcumin on laying performance, egg quality, endocrine hormones, and immune activity in heat-stressed hens. Poult. Sci..

[20] Surai P.F., Kochish I.I., Fisinin V.I., Kidd M.T. (2019). Antioxidant Defence Systems and Oxidative Stress in Poultry Biology: An Update. Antioxidants.

[21] Geng X., Yang L., Hou Y., Zhang Z., He F., Xu R., Zhang P., Jiang R., Li W., Sun G. (2026). Effects of Replacing Soybean Meal with Different Proportions of Black Soldier Fly Larvae Meal on Antioxidant Indicators, Immune System, and Gut Health of Xichuan Black-Bone Chickens. Antioxidants.

[22] Zhao F., Qu J., Wang W., Li S., Xu S. (2020). The imbalance of Th1/Th2 triggers an inflammatory response in chicken spleens after ammonia exposure. Poult. Sci..

[23] Chen Y., Liu L., Yu L., Li S., Zhu N., You J. (2024). Curcumin Supplementation Improves Growth Performance and Anticoccidial Index by Improving the Antioxidant Capacity, Inhibiting Inflammatory Responses, and Maintaining Intestinal Barrier Function in Eimeria tenella-Infected Broilers. Animals.

[24] Zhang J., Zhang R., Jin S., Feng X. (2024). Curcumin, a plant polyphenol with multiple physiological functions of improving antioxidation, anti-inflammation, immunomodulation and its application in poultry production. Anim. Nutr..

[25] Bai D., Hu Y., Liu Y., Xue M., Liu X. (2026). Curcumin alleviates the effects of heat stress on broiler pectoral muscle via decorin (DCN) -mediated TGF-β Signaling pathway. Poult. Sci..

[26] Liu Y., Song M., Bai H., Wang C., Wang F., Yuan Q. (2024). Curcumin improves the egg quality, antioxidant activity, and intestinal microbiota of quails during the late laying period. Poult. Sci..

[27] Moneim A.M.E.A., Mesalam N.M., Yang B., Elsadek M.F. (2025). Dietary incorporation of biological curcumin nanoparticles improved growth performance, ileal architecture, antioxidative status, serum lipid profile, and humoral immune response of heat-stressed broiler chickens. Poult. Sci..

[28] Lin Y., Gong J., Buttimer C., Pan X., Jia Y., Bai Z., Wang R., Tong H., Bao H. (2025). Effects of astaxanthin on growth performance, intestinal integrity, and microbiota in Salmonella Enteritidis-infected chickens. Poult. Sci..

[29] Tan H., Zhen W., Bai D., Liu K., He X., Ito K., Liu Y., Li Y., Zhang Y., Zhang B. (2023). Effects of dietary chlorogenic acid on intestinal barrier function and the inflammatory response in broilers during lipopolysaccharide-induced immune stress. Poult. Sci..

[30] Chen X., Li F., Xu X., Wang L., Yu Y., Yan J., Shan X., Zhang R., Xing H., Zhang T. (2024). Dietary curcumin supplementation enhances growth performance and anti-inflammatory functions by modulating gut microbiota, microbiota-derived metabolites, and expression of inflammation-related genes in broilers. J. Anim. Sci..

[31] Guo S., Hu J., Ai S., Li L., Ding B., Zhao D., Wang L., Hou Y. (2023). Effects of Pueraria Extract and Curcumin on Growth Performance, Antioxidant Status and Intestinal Integrity of Broiler Chickens. Animals.

[32] Nissen L., Casciano F., Chiarello E., Di Nunzio M., Bordoni A., Gianotti A. (2021). Colonic In Vitro Model Assessment of the Prebiotic Potential of Bread Fortified with Polyphenols Rich Olive Fiber. Nutrients.

[33] Meimandipour A., Shuhaimi M., Soleimani A.F., Azhar K., Hair-Bejo M., Kabeir B.M., Javanmard A., Muhammad Anas O., Yazid A.M. (2010). Selected microbial groups and short-chain fatty acids profile in a simulated chicken cecum supplemented with two strains of Lactobacillus. Poult. Sci..

[34] Martín R., Rios-Covian D., Huillet E., Auger S., Khazaal S., Bermúdez-Humarán L.G., Sokol H., Chatel J.M., Langella P. (2023). Faecalibacterium: A bacterial genus with promising human health applications. FEMS Microbiol. Rev..

[35] Li J., Xu J., Guo X., Xu H., Huang C., Nie Y., Zhou Y. (2025). Odoribacter splanchnicus—A Next-Generation Probiotic Candidate. Microorganisms.

[36] Ceylan N., Yenice E., Yavaş İ., Çenesiz A.A., Toprak N.N., Çiftçi İ. (2023). Comparative effects of medium-chain fatty acids or phytobiotics-based feed additives on performance, caecum microbiota, volatile fatty acid production and intestinal morphology of broilers. Vet. Med. Sci..

[37] Rychlik I. (2020). Composition and Function of Chicken Gut Microbiota. Animals.

[38] Syed M.A., Ullah H., Tabassum S., Fatima B., Woodley T.A., Ramadan H., Jackson C.R. (2020). Staphylococci in poultry intestines: A comparison between farmed and household chickens. Poult. Sci..

[39] Wang M., Zhong J., Guo Y., Zhao S., Xia H., Wang G., Liu C., Guo A. (2024). Effects of Adding Sphingomonas Z392 to Drinking Water on Growth Performance, Intestinal Histological Structure, and Microbial Community of Broiler Chickens. Animals.

[40] Salvi P.S., Cowles R.A. (2021). Butyrate and the Intestinal Epithelium: Modulation of Proliferation and Inflammation in Homeostasis and Disease. Cells.

[41] Zhang H., Wang Y., Wang Y., Wei B., Wang L., Nguyen M.T., Lv X., Huang Y., Chen W. (2024). Fermented calcium butyrate supplementation in post-peak laying hens improved ovarian function and tibia quality through the “gut-bone” axis. Anim. Nutr..

[42] Hernández-García P.A., Granados-Rivera L.D., Orzuna-Orzuna J.F., Vázquez-Silva G., Díaz-Galván C., Razo-Ortíz P.B. (2025). Meta-Analysis of Dietary Curcumin Supplementation in Broiler Chickens: Growth Performance, Antioxidant Status, Intestinal Morphology, and Meat Quality. Antioxidants.

[43] Zhai S.S., Ruan D., Zhu Y.W., Li M.C., Ye H., Wang W.C., Yang L. (2020). Protective effect of curcumin on ochratoxin A–induced liver oxidative injury in duck is mediated by modulating lipid metabolism and the intestinal microbiota. Poult. Sci..

[44] Liang S., Lu L., Hong-Fang J. (2017). Regulative effects of curcumin spice administration on gut microbiota and its pharmacological implications. Food Nutr. Res..

[45] Peterson C.T., Vaughn A.R., Sharma V., Chopra D., Mills P.J., Peterson S.N., Sivamani R.K. (2018). Effects of Turmeric and Curcumin Dietary Supplementation on Human Gut Microbiota: A Double-Blind, Randomized, Placebo-Controlled Pilot Study. J. Evid. Based Integr. Med..

[46] Szőke Z., Gashi N., Dávid P., Fauszt P., Mikolás M., Szilágyi-Tolnai E., Szilágyi E., Bíróné Molnár P., Pesti-Asbóth G., Homoki J.R. (2026). Comparative Analysis of Gut Microbiome Composition and Blood Lipid Profiles in Intensively Reared Broiler Chickens and Ducks. Animals.

[47] Oke O.E., Fasasi L.O., Opowoye I.O., Akosile O.A. (2025). The role of gut microbiota-derived metabolites in modulating poultry immunometabolism. Front. Physiol..

[48] Wang T., Cheng K., Li Q., Wang T. (2022). Effects of yeast hydrolysate supplementation on intestinal morphology, barrier, and anti-inflammatory functions of broilers. Anim. Biosci..

[49] Wang J., Wu Y., Zhou T., Feng Y., Li L.A. (2025). Common factors and nutrients affecting intestinal villus height-A review. Anim. Biosci..

[50] Liu H., Meng H., Du M., Lv H., Wang Y., Zhang K. (2024). Chlorogenic acid ameliorates intestinal inflammation by inhibiting NF-κB and endoplasmic reticulum stress in lipopolysaccharide-challenged broilers. Poult. Sci..

[51] Byun J.S., Jeong W.I. (2010). Involvement of hepatic innate immunity in alcoholic liver disease. Immune Netw..

[52] Xu Q., Guo M., Wang H., Liu H., Wei Y., Wang X., Mackay C.R., Wang Q. (2024). A Butyrate-Yielding Dietary Supplement Prevents Acute Alcoholic Liver Injury by Modulating Nrf2-Mediated Hepatic Oxidative Stress and Gut Microbiota. Int. J. Mol. Sci..

[53] Lee C., Kim B.G., Kim J.H., Chun J., Im J.P., Kim J.S. (2017). Sodium butyrate inhibits the NF-kappa B signaling pathway and histone deacetylation, and attenuates experimental colitis in an IL-10 independent manner. Int. Immunopharmacol..

