# Supplementary material for: Effects of Curcumin Supplementation on Production Performance, Intestinal Health and Cecal Microbiota in Late-Phase Jingfen No. 1 Laying Hens
Source: Animals (Basel). 2026 Oct 29;16(21):3415. doi: 10.3390/ani16213415 (PMC13762402; doi:10.3390/ani16213415)
Supplement: Supplementary file 1 [file animals-16-03415-s001.zip › animals-4586159-supplementary.pdf]

Supplementary Materials

Table S1. Baseline production performance of Jingfen No. 1 laying hens at 60 weeks of age before curcumin supplementation

Items	Groups				SEM	*P*-value
	CG	CN1	CN2	CN3		Anova
ADFI, g/d	114.84	122.34	116.60	117.29	10.15	0.642
EPR, %	77.57	74.60	73.58	75.67	5.77	0.694
AEW, g	55.05	56.83	53.78	54.53	4.62	0.727
FER	2.72	2.89	2.98	2.87	0.38	0.706

ADFI, average daily feed intake; EPR, egg production rate; AEW, average egg weight; FER, feed-to-egg ratio; SEM, standard error of the mean. All $P > 0.05$. No significant differences were observed among groups at baseline.

Table S2. Weekly production performance of late-laying hens supplemented with graded levels of curcumin.

Items	Groups				SEM	P-value		
	CG	CN1	CN2	CN3		ANOVA	Linear	Quadratic
Weeks 1								
ADFI, g/d	111.54	115.04	110.16	121.27	4.10	0.254	0.134	0.379
EPR, %	63.55	59.27	59.75	63.76	1.94	0.234	0.638	0.057
AEW, g	51.88	53.16	53.56	53.68	1.03	0.603	0.266	0.464
FER	3.40	3.66	3.45	3.58	0.15	0.590	0.609	0.764
Weeks 2								
ADFI, g/d	113.47	113.21	113.49	122.12	4.00	0.338	0.112	0.385
EPR, %	62.56	56.96	59.20	62.97	2.12	0.175	0.502	0.065
AEW, g	52.25	55.01	55.00	56.89	1.09	0.051	0.011	0.478
FER	3.48	3.63	3.50	3.47	0.17	0.894	0.782	0.707
Weeks 3								
ADFI, g/d	113.09	113.72	108.56	123.60	4.19	0.110	0.091	0.113
EPR, %	63.64	55.78	60.13	63.31	2.26	0.080	0.530	0.052
AEW, g	51.09^{b}	56.94^{a}	55.87^{a}	58.85^{a}	1.26	0.002	0.001	0.159
FER	3.48	3.59	3.26	3.38	0.17	0.532	0.451	0.703
Weeks 4								
ADFI, g/d	111.94	114.86	110.54	123.73	4.48	0.190	0.085	0.306
EPR, %	60.54	55.81	59.00	64.15	2.13	0.077	0.088	0.077
AEW, g	52.89^{b}	56.87^{ac}	56.30^{c}	59.90^{a}	1.13	0.003	<0.001	0.567
FER	3.51	3.66	3.33	3.27	0.19	0.452	0.217	0.854
Weeks 5								
ADFI, g/d	114.97	113.17	112.26	124.28	3.67	0.111	0.060	0.113
EPR, %	60.26	54.09	58.12	62.01	2.11	0.080	0.214	0.068
AEW, g	52.75^{b}	59.13^{ac}	57.98^{c}	62.43^{a}	1.13	<0.001	<0.001	0.190
FER	3.63	3.55	3.34	3.25	0.15	0.250	0.058	0.695

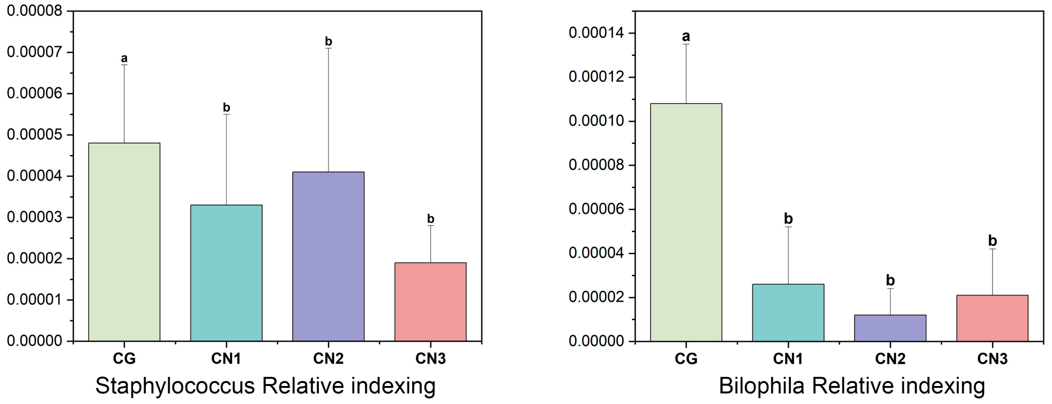

Figure S 1. Pathogen grading chart.
